# BCLAF1 binds SPOP to stabilize PD-L1 and promotes the development and immune escape of hepatocellular carcinoma

**DOI:** 10.1007/s00018-024-05144-z

**Published:** 2024-02-10

**Authors:** Zongdong Yu, Xiang Wu, Jie Zhu, Huan Yan, Yuxuan Li, Hui Zhang, Yeling Zhong, Man Lin, Ganghui Ye, Xinming Li, Jiabei Jin, Kailang Li, Jie Wang, Hui Zhuang, Ting Lin, Jian He, Changjiang Lu, Zeping Xu, Xie Zhang, Hong Li, Xiaofeng Jin

**Affiliations:** 1 Department of Hepatobiliary and Pancreatic Surgery, Affiliated Li Huili Hospital, Ningbo University, Ningbo, 315040 China; 2 Department of Biochemistry and Molecular Biology, and Zhejiang Key Laboratory of Pathophysiology, Health Science Center, Nngbo University, Ningbo, 315211 China

**Keywords:** BCLAF1, SPOP, PD-L1, Immunotherapy, Hepatocellular carcinoma

## Abstract

Interaction between programmed death-1 (PD-1) ligand 1 (PD-L1) on tumor cells and PD-1 on T cells allows tumor cells to evade T cell-mediated immune surveillance. Strategies targeting PD-1/PD-L1 have shown clinical benefits in a variety of cancers. However, limited response rates in hepatocellular carcinoma (HCC) have prompted us to investigate the molecular regulation of PD-L1. Here, we identify B cell lymphoma-2-associated transcription factor 1 (BCLAF1) as a key PD-L1 regulator in HCC. Specifically, BCLAF1 interacts with SPOP, an E3 ligase that mediates the ubiquitination and degradation of PD-L1, thereby competitively inhibiting SPOP-PD-L1 interaction and subsequent ubiquitination and degradation of PD-L1. Furthermore, we determined an SPOP-binding consensus (SBC) motif mediating the BCLAF1-SPOP interaction on BCLAF1 protein and mutation of BCLAF1-SBC motif disrupts the regulation of the SPOP-PD-L1 axis. In addition, BCLAF1 expression was positively correlated with PD-L1 expression and negatively correlated with biomarkers of T cell activation, including CD3 and CD8, as well as with the level of immune cell infiltration in HCC tissues. Besides, BCLAF1 depletion leads to a significant reduction of PD-L1 expression in vitro, and this reduction of PD-L1 promoted T cell-mediated cytotoxicity. Notably, overexpression of BCLAF1 sensitized tumor cells to checkpoint therapy in an in vitro HCC cells-Jurkat cells co-culture model, whereas BCLAF1-SBC mutant decreased tumor cell sensitivity to checkpoint therapy, suggesting that BCLAF1 and its SBC motif serve as a novel therapeutic target for enhancing anti-tumor immunity in HCC.

**Supplementary Information:**

The online version contains supplementary material available at 10.1007/s00018-024-05144-z.

## Introduction

An important component of T cell-mediated tumor elimination is the interaction between T cell receptors (TCRs) on T cells and peptide major histocompatibility complexes (MHCs) on target cells [1]. Nonetheless, this process is regulated primarily by numerous co-suppressive and co-stimulatory ligands and their receptors known as immune checkpoints [1]. Of these, the PD-1/PD-L1 axis has emerged as a prominent therapeutic target in many malignancies. More than 1000 clinical trials have demonstrated the extensive clinical advantages of immune checkpoint blockade (ICB) therapies focused on the PD-1/PD-L1 axis [2]. At present, the combination of the anti-PDL1 antibody Atezolizumab and the vascular endothelial growth factor (VEGF) neutralizing antibody Bevacizumab has become the first-line treatment for patients with advanced HCC [3]. Although PD-1/PD-L1 blockade has produced striking improvements in clinical outcomes for patients with cancers, only a minority of HCC patients have shown a durable response to these therapies, and intrinsic resistance remains a huge challenge. It has been shown that PD-L1 expression on tumor cells, tumor mutational load, and T lymphocyte infiltration may be key indicators of clinical response [4]. PD-L1 expression status appears to be particularly important. Upregulated PD-L1 usually contributes to cancer immune escape, which ultimately leads to cancer progression [5, 6]. Moreover, The National Comprehensive Cancer Network has guidelines stating that PD-L1 expression is the primary indicator of ICB therapy for some cancers, such as non-small-cell lung cancer. Therefore, it is crucial to continuously examine the regulatory process of PD-L1 expression that can improve the efficacy of ICB therapy in HCC.

Studies have indicated that PD-L1 expression was regulated by transcriptional, post-transcriptional, and post-translational mechanisms [1, 7, 8]. Among them, post-translational modification plays a crucial role in regulating PD-L1 stability, activation, localization, and interaction [9]. Abnormal patterns of ubiquitination modification are involved in PD-L1 upregulation in the tumor microenvironment (TME) [10]. It has been shown that CKLF-like MARVEL transmembrane domain protein 6 (CMTM6) maintains PD-L1 expression by decreasing ubiquitination of PD-L1 and prolonging its half-life in a wide range of cancer cells[10]. Besides, the TNF-α-NF-κB pathway inhibited the ubiquitination of PD-L1 by up-regulating COP1 signalosome 5 (CSN5) [11]. Restoring PD-L1 expression via the inhibition of CSN5 sensitized tumor cells to subsequent immunotherapies [11]. In addition, the Cullin 3-SPOP E3 ligase promotes PD-L1 ubiquitination via cell cycle protein D/cell cycle protein-dependent kinase 4 (CDK4) [12]. Treatment with CDK4/6 inhibitors enhances PD-L1 abundance, thereby suggesting potential for combinational use with α-PD-1/PD-L1 drugs [12]. These findings suggest that ubiquitination modification of PD-L1 may be a potential targeting process to improve the efficacy of ICB therapy.

Notably, Speckle-type POZ protein (SPOP) serves as a tumor suppressor through its function as a substrate receptor for Cullin 3 (CUL3)-based ubiquitin ligases and mediates ubiquitination and degradation of PD-L1 [12]. Not limited to the post-translational level, Gao et al*.* [13] reported that SPOP negatively regulates PD-L1 expression at the transcriptional level. Specifically, SPOP binds to IRF1, a major transcription factor that induces PD-L1 expression, and subsequently triggers ubiquitin-proteasomal degradation of IRF1 to inhibit IRF1-mediated upregulation of PD-L1 transcription [13]. Thus, SPOP performs a unique role in regulating the homeostatic levels of PD-L1/PD-1 signaling in cancer cells, including prostate cancer, colorectal cancer, and esophageal adenocarcinoma [12, 14–16]. Furthermore, abnormal SPOP-mediated ubiquitination modifications lead to the development of HCC [17]. Several studies have found that SPOP is lowly expressed in HCC compared to normal liver tissue and represses the proliferation and migration of HCC cells [18, 19]. Further mechanistic studies suggest that SPOP inhibits HCC cell metastasis through ubiquitin-dependent degradation of SUMO1/sentrin-specific peptidase 7 (SENP7) [20]. Although multiple downstream effectors of SPOP have been identified, little is known about the upstream regulatory mechanisms that may influence the tumor suppressive function of SPOP in HCC.

B cell lymphoma-2-associated transcription factor 1 (BCLAF1) was initially identified as a regulator of apoptosis and transcription and since then has been shown to be involved in many biological processes, such as splicing and preprocessing of mRNA, DNA damage response, autophagy, T cell activation, muscle cell proliferation and differentiation, lung development, ischemia–reperfusion injury, and viral infection [21]. Emerging evidence suggested that BCLAF1 is involved in the regulation of angiogenesis, cell proliferation, and drug resistance in HCC by activating the transcription of hypoxia-inducible factor-1α (HIF-1α) [22–24]. It has also been shown that Hsp90α-dependent BCLAF1 protects oncogene *c-MYC* mRNA from degradation through its RS structural domain, thereby promoting HCC occurrence and progression [25]. Here, we found that BCLAF1 interacts with SPOP and regulates PD-L1 expression. This leads us to propose that BCLAF1 potentially plays a role in immune surveillance via SPOP-PD-L1 axis.

In this work, we demonstrate that BCLAF1 competitively inhibits SPOP-mediated ubiquitination and degradation of PD-L1 by interacting with SPOP to maintain PD-L1 expression, ultimately promoting immune evasion and tumor progression of HCC. Furthermore, we confirmed the primary motifs of BCLAF1-SPOP interaction, and we also identified BCLAF1 as a potential therapeutic target and the efficacy of ICB treatment could be increased in HCC with high expression of BLCAF1 in vitro.

## Materials and methods

Antibody, chemicals, primers, and siRNA/sh RNA sequence information used in this study are listed in Supplemental Tables 5–7.

### Cell culture, transfection, and lentivirus infection

For cell culture, HEK-293T and Jurkat cell lines was obtained from Haixing Biosciences (Suzhou, Jiangsu, China). The hepatocellular carcinoma cell lines (HepG2 and SK-Hep1) were obtained from Procell Life Science & Technology (Wuhan, Hubei, China). HEK-293T and SK-Hep1 cells were cultured in Dulbecco's Modified Eagle Medium (DMEM, Meilunbio, China) with 10% Fetal Bovine Serum (FBS, Standard Quality, OriCell, China). Jurkat cells were cultured in Roswell Park Memorial Institute-1640 (RPMI-1640, Meilunbio, China) with 10% Fetal Bovine Serum (FBS, Standard Quality, OriCell, China). HepG2 cells were cultured in Minimum Essential Medium (MEM, Meilunbio, China) with 10% Fetal Bovine Serum (Standard Quality, OriCell, China). All cells were grown at 37 °C with 5% CO_2_. For transfection, cells were transiently transfected with plasmid, siRNA, and shRNA using Lipo6000 Transfection Reagent (Beyotime, Shanghai, China) according to the manufacturer's protocol. For lentivirus infection, lentivirus containing pLVX-BCLAF1-Puro, pLVX-shBCLAF1-Puro, pLVX-SPOP-Puro, pLVX-shSPOP-Puro, and corresponding Control group lentivirus were purchased from Obio Technology (Shanghai, China) and were used to infect HepG2 and SK-Hep1 cells according to the manufacturer's protocol. Infected cells were then subjected to puromycin selection (5 μg/mL), and stable transfection of cells was confirmed by western blot.

### Bioinformatic analysis

For The Cancer Genome Atlas (TCGA) database (https://portal.gdc.cancer.gov/), we extracted and downloaded a LIHC dataset by R software (version 4.2.1) and performed differential expression analysis, clinicopathological characterization, prognostic analysis, ROC curve analysis, correlation analysis, and response to immunotherapy analysis. For the GEO database (http://www.ncbi.nlm.nih.gov/geo/), we extracted and downloaded the HCC dataset (GSE14520) via R software (version 4.2.1) and performed differential expression analysis on the mRNA transcript data of BCLAF1 contained in this dataset. For the CPTAC database (https://proteomics.cancer.gov/programs/cptac/), we extracted and downloaded the LIHC dataset via R software (version 4.2.1) and performed differential expression analysis on the protein data of BCLAF1 contained in this dataset.

In addition, we analyzed the correlation between BCLAF1 expression and tumor grade and stage in HCC patients using the UALCAN website (http://ualcan.path.uab.edu/) [26]; the GEPIA 2 (http://gepia2.cancer-pku.cn/) [27] and TIMER 2.0 (http://timer.cistrome.org/) [28] websites to determine the correlation between BCLAF1 and PD-L1; the TIMER 2.0 website to determine the relationship between BCLAF1 expression and immune cell infiltration [28]; the GRTD (http://gtrd.biouml.org/) [29], HumanTFDB (http://bioinfo.life.hust.edu.cn/HumanTFDB/) [30], and PROMO (https://alggen.lsi.upc.es/cgi-bin/promo_v3/promo/promoinit.cgi?dirDB=TF_8.3/) [31] websites to predict potentially ten transcription factors targeting BCLAF1; the miRDB (https://mirdb.org/) [32], mirDIP (http://ophid.utoronto.ca/mirDIP/index_confirm.jsp/) [33], miRWalk (http://mirwalk.umm.uni-heidelberg.de/) [34] and TargetScan (https://www.targetscan.org/vert_72/) [35] websites to predict potentially five microRNAs (miRNAs) targeting BCLAF1; the ubibrowser website (http://ubibrowser.bio-it.cn/ubibrowser/) to predicted potentially twenty E3 ubiquitin ligases targeting BCLAF1 [36].

### Plasmid constructions

Expression vectors for SPOP have been described previously [37]. BCLAF1 cDNA sequences were subcloned into pCMV-Flag and pCMV-Myc expression vectors. Flag-PD-L1 overexpression plasmid was kindly provided by Dr. Xiangguo Liu (College of Life Sciences, Shandong University, China). BCLAF1 mutants were generated using the KOD-Plus-Mutagenesis kit (TOYOBO) according to the manufacturer's instructions. All constructs were validated by DNA sequencing.

### Western blot

Cells were lysed with RIPA lysis buffer (High) supplemented with protease inhibitors on ice for 30 min, lysates or immunoprecipitates were subjected to SDS-PAGE, and proteins were transferred to nitrocellulose membranes (GE Healthcare Sciences). Membranes were closed in Tris-buffered saline (TBS, pH 7.4) containing 5% skim milk and 0.1% Tween 20, washed twice in TBS containing 0.1% Tween 20, and incubated overnight at 4 °C with primary antibody, followed by the secondary antibody for 1 h at room temperature. The target protein was visualized using an enhanced chemiluminescence (ECL) system (Santa Cruz Biotechnology). WB was performed 2–3 times from at least two independent experiments and representative images are shown.

### In vivo ubiquitination assays and co-immunoprecipitation (Co-IP)

HEK-293T or SK-Hep1 cells were transfected with HA-ubiquitin and/or the indicated constructs. 36 h after transfection, cells were treated with MG-132 (20 μM) for 8 h before harvesting, then lysed in RIPA lysate (High) and boiled for 10 min. For Co-IP, the WCLs were centrifuged at 12,000 × rpm for 20 min. The supernatant was removed and incubated with anti-Flag M2 agarose beads (Sigma, USA) or with recombinant protein G sepharose beads (Saier, China) coupled with PD-L1 antibody at 4 °C overnight. The bound beads are then washed three times with BC100 buffer (20 mM Tris–Cl, pH 7.9, 100 mM NaCl, 0.2 mM EDTA, 20% glycerol) containing 0.2% Triton X-100. Proteins were eluted with Flag peptide for 4 h at 4 °C or by boiling in SDS-PAGE solution. The ubiquitinated forms of BCLAF1 and PD-L1 as well as the immunoprecipitated pull-down proteins were detected by Western blot using anti-HA antibody, anti-ubiquitin antibody, or anti-PD-L1 antibody coupled with other labeling antibodies.

### GST pull-down assay

GST fusion proteins were immobilized on glutathione–sepharose beads (Amersham Biosciences, USA). The beads were washed using pull-down buffer (20 mM Tris–HCl pH 7.5, 150 mM NaCl, 0.1% NP-40, 1 mM DTT, 10% glycerol, 1 mM EDTA, 2.5 mM MgCl_2_, and 1 μg/mL leupeptin). The beads were incubated with recombinant protein tagged with His for two hours before being washed five times with binding buffer. Finally, the beads were resuspended in a sample buffer and the bound proteins were subjected to SDS-PAGE and Western blot analysis.

### Proximity ligation assay (PLA)

SK-Hep1 cells were seeded in 24-well chamber slides and incubated in DMEM for 24 h. Subsequently, cells were fixed with 4% paraformaldehyde and permeabilized with 0.4% Triton X-100, and blocked with Duolink Blocking buffer (Sigma, USA) for 1 h at 37 °C. In situ PLA was performed using the Duolink in situ Red kit (Sigma-Aldrich, USA). Primary antibodies, including anti-BCLAF1 and anti-SPOP, were incubated overnight at 4 °C. On the following day, the secondary antibody was incubated at 4 °C for 1 h, and the Plus and Mines PLA probes were incubated at 37 °C for 1 h. The Duolink In Situ Detection Reagents Red (Sigma, USA) was used to perform ligation and amplification of the PLA. After multiple washes, cells were mounted in Prolong Gold mounting media with DAPI and imaged using a confocal microscope (LSM880, Zeiss, Japan) with a 63*/1.4NA Oil PSF Objective.

### Cell immunofluorescence

For cell immunofluorescence, cells were placed on chamber slides and fixed in 4% paraformaldehyde for 30 min at room temperature. After washing with PBS, the cells were permeabilized with 0.1% Triton X-100 in PBS for 15 min. Cells were then washed with PBS, blocked with 0.5% BSA in PBS for 1 h, and incubated with the primary antibody in PBS overnight at 4 °C. The cells were then washed with PBS, blocked with 0.5% BSA in PBS, and incubated with the primary antibody in PBS overnight at 4 °C. After washing with PBS, fluorescently labeled secondary antibodies were applied and the DAPI was re-stained for 1 h at room temperature. Cells were visualized and imaged using fluorescent microscope (Nikon Ds-Ri2, Japan).

### Quantitative real-time reverse transcription PCR (qRT-PCR)

Total RNA was isolated from ECC-1 and HEC-1-A cells using TRIzol reagent (Tiangen, China), and cDNA was reverse-transcribed using HiScript® II 1st Strand cDNA Synthesis Kit (Vazyme, China) according to the manufacturer's instructions. PCR amplification was performed using SYBR Green PCR Master Mix Kit (Vazyme, China). All quantifications were normalized to the levels of the endogenous Control group GAPDH.

### Cycloheximide treatment

To measure the effect of BCLAF1 and/or SPOP on PD-L1 protein stability, HepG2 and SK-Hep1 cells transfected with the indicated plasmids were treated with the protein synthesis inhibitor CHX (50 μg/mL) prior to collection, followed by Western blot.

### Tissue samples and immunohistochemistry

Archived formalin-fixed, paraffin-embedded hepatocellular carcinoma (HCC) samples were collected from patients diagnosed with primary hepatocellular liver cancer after radical HCC resection at Affiliated LiHuiLi Hospital of Ningbo University from January 2020 to August 2022. Malignant disease or having received preoperative treatment (chemotherapy and/or radiotherapy) was excluded from this study. The retrieval of tissue and clinical data was approved by the Institutional Review Board of Ethics of Ningbo University (NBU-2022-123).

A total of 105 pairs of HCC tissues were cut to 4 μm thickness, heated at 65 °C for 2 h, dewaxed in xylene, and rehydrated in a series of graded ethanol. Antigen repair was performed by heating tissue sections in ethylenediaminetetraacetic acid buffer (pH 9.0) for 10 min using a pressure cooker and then cooling to room temperature. After 30 min with peroxidase blocking reagent (3% H_2_O_2_ solution), tissues are washed 3 times with PBST solution and incubated overnight at 4 °C in a humidified chamber with PD-L1 primary antibody (Proteintech, #66248-1-Ig, Wuhan, China) or BCLAF1 primary antibody (Proteintech, #67860-1-Ig, Wuhan, China). After washing the tissue Sections 3 times with PBST, they were incubated with HRP polymeric anti-mouse/rabbit secondary antibody incubated for 30 min. The antibody assay was visualized using the DAB assay kit (Solarbio, #G1212, Beijing, China) to visualize the antibody detection. Slides were re-stained with hematoxylin.

All samples were reviewed by two independent pathologists experienced in the evaluation of IHC who did not know the clinical outcome of these patients. We assessed the percentage of positively stained cells and the intensity of staining to determine BCLAF1 and PD-L1 expression semi-quantitatively. The percentage of positively staining cells was scored as follows: 0, < 10%; 1, 10%–50%; and 2, > 50%. The intensity of staining was graded as follows: 0 (no or weak staining = light yellow), 1 (moderate staining = yellowish brown), and 2 (strong staining = brown). The total score for BCLAF1 and PD-L1 expression was the sum of the percentage of cells scored against positive staining and the intensity of staining score, and a total score from 0 to 4 was assigned [38–40]. For statistical analysis, the final score was a combination of the independent scores assigned by the two pathologists reported in this study. Any differences in scores were resolved by discussion between the two pathologists.

### Cell proliferation assay

The cell proliferation rate was determined using Cell Counting Kit-8 (CCK-8) (Dojindo Laboratories, Japan) according to the manufacturer's protocol. Briefly, cells were inoculated onto 96-well plates at a density of 1,000 cells per well. During the incubation period of 0–6 days, 10 μl of CCK-8 solution was added to the cell cultures and incubated for 2 h. The OD value of each well was measured at 450 nm using a microplate absorbance reader (Bio-Rad, US). Each assay was performed in triplicate.

### Colony formation assay

HepG2 and SK-Hep1 cells were inoculated in 6-well plates containing 1500 individual cells per well in triplicate. After 2 weeks of incubation, the cells were fixed in 100% methanol for 5 min at room temperature and then stained with Giemsa dye for 20 min (Solarbio, China).

### Wound-healing assay

HepG2 and SK-Hep1 cells were inoculated in 6-well plates (Costar, Corning, US) and cultured to 80% fusion. Monolayers of cells were damaged by removing the culture insert and rinsed with PBS to remove cellular debris. After treatment with Mitomycin C for 1 h (5 μM) (GLPBIO, #GC12353, CA, USA), the medium was replaced with fresh serum-free DMEM. After 48 h of migration, cells were stained with 0.2% crystal violet for 20 min at room temperature. Images were acquired using fluorescent microscope (Nikon Ds-Ri2, Japan) at 0 h and 48 h after migration. The area of wound edge healing was calculated between 0 and 48 h.

### Migration and invasion assays

HepG2 and SK-Hep1 cells were precultured in a serum-free medium for 48 h. For the migration assay, 4 × 10^4^ cells were inoculated into the upper side of a modified Boyden chamber (8.0 μm, #3342, Corning, NY, USA) and the lower chamber was filled with medium containing 5% FBS. After 24 h, we carefully removed non-migrating cells from the upper chamber with a cotton swab and stained and counted migrating cells in nine different areas below the filter. Matrix gel invasion assays were performed using migration inserts (Costar) coated with matrix gel/fibronectin (BD Biosciences, USA). Photographs of the stained cells were taken with a microscope (magnification: 200×).

### HCC and Jurkat cells co-culture system

HepG2 and SK-Hep1 cells with BCLAF1 and/or SPOP stably knockdown/overexpressed were inoculated into 12-well plates at a density of 1 × 10^5^, transfected with exogenous overexpression of plasmids or siRNAs, and the medium was replaced with fresh medium after 24 h. Jurkat cells were pre-activated for 24 h with 2 μg/mL of soluble human CD3/CD28 T cell activator ( Proteintech, #KMS310, Wuhan, China). Jurkat cells were then mixed with HepG2 and SK-Hep1 as well as control group cells at a density of 5 × 10^5^, and DMSO or Atezolizumab (10 ng/ml) was also added for co-cultivation. 24 h later, the culture medium was collected to ELISA assay for IL-2, IL-4, IL10, and IFN-γ and the Jurkat cells for flow cytometry analysis.

### Flow cytometry

Flow cytometry was performed using Annexin V-FITC/PI Apoptosis Kit (Multi Sciences, #AP101, Hangzhou, China) and FITC/PI Cell Cycle Staining Kit (Multi Sciences, #CCS012, Hangzhou, China), respectively, according to the manufacturer's instructions and the data were analyzed using FlowJo software (v10.8.1).

### PD-L1/PD-1 binding assay

Cells (1 × 10^6^) were incubated with 5 μg/ml of recombinant human PD-1 FC chimera protein (#1086-PD-050, R&D Systems, USA) for 30 min at room temperature. After washing with staining buffer, the cells were incubated with an anti-human Alexa Fluor 488 dye-conjugated antibody (ThermoFisher Scientific, USA) for an additional 30 min at room temperature. The cells were then analyzed by flow cytometry after being washed again with staining buffer. The flow cytometry data were analyzed utilizing FlowJo software (v10.8.1), with the cutoff line for relative positive percentage set at the median of the maximum signal.

### ELISA

Cytokine assays were performed using Human IL-2 (#EK102-48), Human IL-4 (#EK104/2-48), Human IL-10 (#EK110/2-48), Human TNF-α (#EK182-48), and Human IFN-γ (#EK180-48) ELISA kits (Multi Sciences, Hangzhou, China).

### Statistical analysis

Statistical calculations were performed using GraphPad Prism software (v8.0), analyze and quantify images using ImageJ software (v1.46). For experiments repeated at least three times, all data are shown as mean ± SD. The differences between the two groups were analyzed through the use of Student's t test, and multiple comparisons were performed using two-way analysis of variance (ANOVA). * represents *p* < 0.05, ** represents *p* < 0.01, *** represents *p* < 0.001, no significance (ns) indicates *P* ≥ 0.05.

## Results

### High expression of BCLAF1 is correlated with poor prognosis of HCC patients

**Fig. 1:**
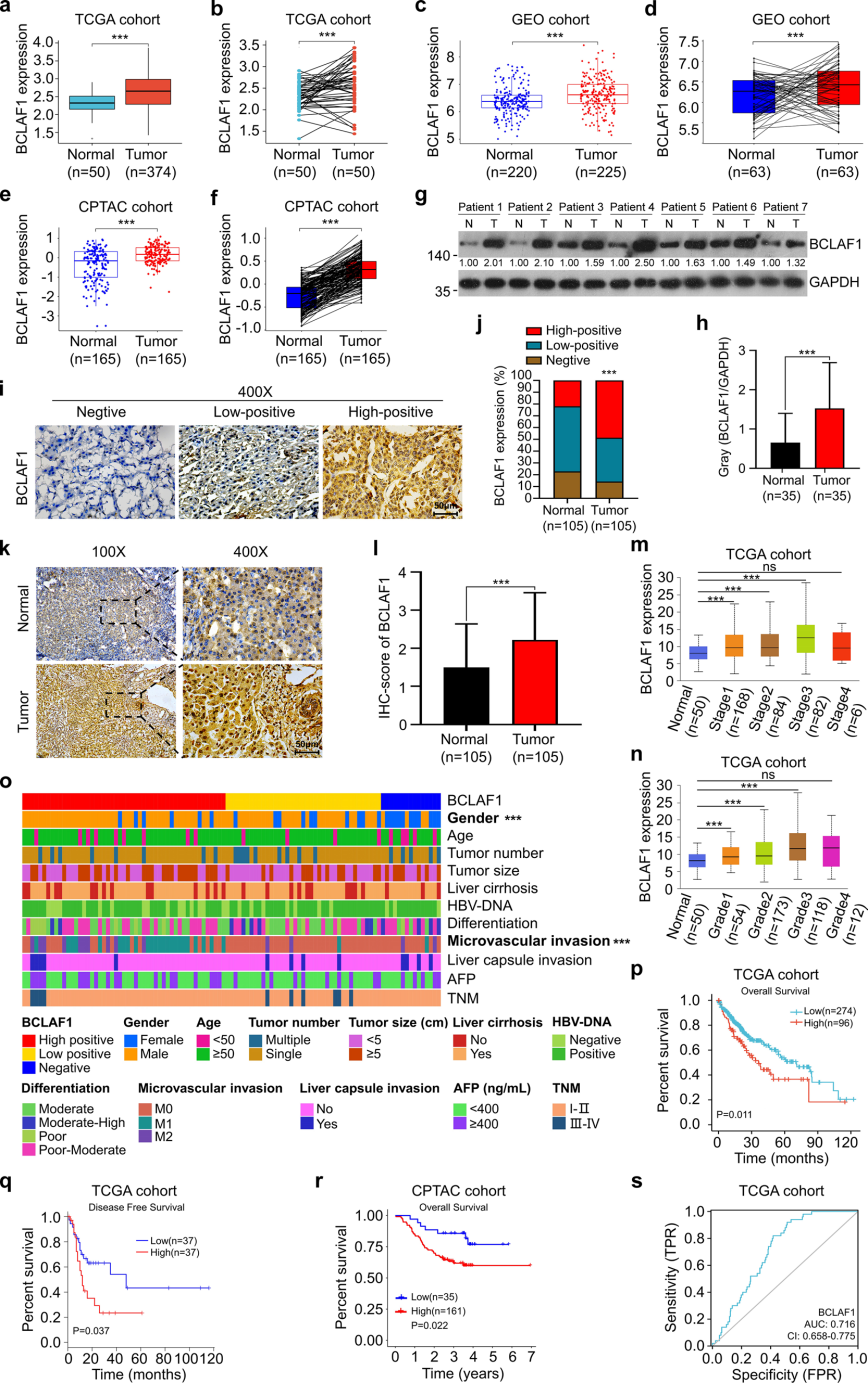
High expression of BCLAF1 is correlated with poor prognosis of HCC patients. **a**, **b** TCGA cohort (LIHC dataset) analyses showed a high mRNA expression levels of BCLAF1 in HCC tissues than in normal liver tissues (**a**) (Normal, *n* = 50; Tumor, *n* = 374) as well as paired normal liver tissues (**b**) (Normal, *n* = 50; Tumor, *n* = 50). **c**, **d** GEO cohort (GSE14520 dataset) analyses showed a high mRNA expression levels of BCLAF1 in HCC tissues than in normal liver tissues (**c**) (Normal, *n* = 220; Tumor, *n* = 225) as well as paired normal liver tissues (**d**) (Normal, *n* = 63; Tumor, *n* = 63). **e**, **f** CPTAC cohort analyses showed a high protein expression of BCLAF1 in HCC tissues than in normal liver tissues (**e**) (Normal, *n* = 165; Tumor, *n* = 165) as well as paired normal liver tissues (**f**) (Normal, *n* = 165; Tumor, *n* = 165). **g** Representative images of BCLAF1 expression of fresh HCC tissues and normal liver tissues were analyzed by western blot (Normal (N), *n* = 35; Tumor (T), *n* = 35). All quantitation was normalized to the protein levels of GAPDH in tumor tissue of each patient. **h** Statistical graph of relative expression of BCLAF1 in HCC tissues in (**g**) (Normal, *n* = 35; Tumor, *n* = 35). **i** IHC demonstrated representative images of the different expression of BCLAF1 in paraffin-embedded HCC tissues and the proportion of 3 different staining intensities (Negative, Low-positive, High-positive). Scale bar, 50 μm. **j** Statistical graph of IHC in (**i**) (Normal, *n* = 35; Tumor, *n* = 35). **k** IHC showed representative images of BCLAF1 expression levels in paraffin-embedded HCC and normal liver tissues. **l** Statistical graph of IHC in **k** (Normal, *n* = 105; Tumor, *n* = 105). Scale bar, 50 μm. **m, n** High expression of BCLAF1 was associated with poorer patient stage (**m**) (Normal, *n* = 50; Tumor, *n* = 340) and tumor grade (**n**) (Normal, *n* = 50; Tumor, *n* = 357) in HCC based on the TCGA cohort (LIHC dataset). **o** Heat map of clinical characteristics analysis of 105 HCC patients. **p**, **q** Survival curves from the TCGA cohort (LIHC dataset) indicated that HCC patients with high mRNA expression levels of BCLAF1 had a short overall survival (**p**) (Low, *n* = 274; High, *n* = 96) and disease-free survival (**q**) (Low, *n* = 37; High, *n* = 37). **r** Survival curves from the CPTAC cohort (LIHC dataset) indicated that HCC patients with a high protein levels of BCLAF1 had a short overall survival (Low, *n* = 35; High, *n* = 161). **s** ROC curve analysis of BCLAF1 based on TCGA HCC data. All data are shown as mean ± SD (n = 3). ***P < 0.001, ns *P* ≥ 0.05

To explore the expression and role of BCLAF1 in the development of HCC, we analyzed the expression of BCLAF1 in HCC and normal liver tissues from The Cancer Genome Atlas (TCGA), Gene Expression Omnibus (GEO), and Clinical Proteomic Tumor Analysis Consortium (CPTAC) databases. Results showed that at the mRNA expression levels, it was higher in HCC tissues compared with normal liver tissues as well as paired normal liver tissues derived from the TCGA database (Fig. 1a, b), and consistent results were obtained based on the analysis of the GEO database (Fig. 1c, d). In addition, at the protein levels, analysis based on the CPTAC database showed elevated BCLAF1 expression in HCC tissues compared with normal liver tissues as well as paired normal liver tissues (Fig. 1e, f). Western blot confirmed higher BCLAF1 expression in HCC tissues than in paired normal liver tissues derived from our collection of 35 pairs of fresh human tissues (Fig. 1g, h). Consistent with this, the results of immunohistochemistry (IHC) of 105 pairs of paraffin-embedded HCC and paired normal liver tissues showed that the expression of BCLAF1 protein was upregulated and highly positively stained in a large percentage of HCC tissues compared with paired normal liver tissues (Fig. 1i–l). Next, correlation analysis of mRNA expression levels of BCLAF1 and clinicopathological features based on the TCGA dataset illustrated that high expression of BCLAF1 in HCC was correlated with advanced stage (Stage III), poorly differentiated grade (Grade 2), and higher alpha-fetoprotein (AFP) levels (Fig. 1m, n and Supplemental Table 1). Analysis of immunohistochemistry results based on 105 paraffin-embedded HCC tissues suggested that HCC patients with high-positive BCLAF1 tissue staining had a higher grade of vascular invasion (M2) and a male gender predisposition (Fig. 1o and Table 1). Moreover, Kaplan–Meier survival analysis using TCGA clinical data indicated that HCC patients with high mRNA expression levels of BCLAF1 had significantly shorter overall survival and disease-free survival compared to patients with low mRNA expression levels of BCLAF1 (Fig. 1p, q). In line with these results, Kaplan–Meier survival analysis based on CPTAC clinical data suggested that HCC patients with high protein expression of BCLAF1 had significantly shorter overall survival than HCC patients with low protein expression of BCLAF1 (Fig. 1r). Besides, the predictive value of BCLAF1 in the diagnosis of HCC was assessed using the receiver operating characteristic (ROC) curve, and the results showed that BCLAF1 has exhibited favorable sensitivity and specificity for the diagnosis of HCC (AUC = 0.716) (Fig. 1s). Together, these findings suggest that BCLAF1 is a risk factor for HCC progression.

**Table 1:** Clinical characteristics of 105 pairs of HCC tissues

Characteristic	Number	BCLAF1			*χ*^2^	*P* value
		Negative	Low positive	High positive		
*n*	105	15	39	51		
Gender					23.00	1E − 05
Male	78	4	29	45		
Female	27	11	10	6		
Age (years)					4.08	0.13
< 50	18	5	4	9		
≥ 50	87	10	35	42		
Tumor number					2.35	0.309
Single	83	10	30	43		
Multiple	22	5	9	8		
Tumor size (cm)					2.06	0.357
< 5	58	8	25	25		
≥ 5	47	7	14	26		
Liver cirrhosis					0.56	0.756
Yes	77	12	29	36		
No	28	3	10	15		
HBV					1.66	0.435
Negative	27	3	8	16		
Positive	78	12	31	35		
Differentiation					7.96	0.219
Poor	12	1	4	7		
Poor–Moderate	44	7	15	22		
Moderate	37	3	14	20		
Moderate–High	12	4	6	2		
Microvascular invasion					28.71	3E-06
M0	62	12	33	17		
M1	30	1	5	24		
M2	13	2	1	10		
Liver capsule invasion					1.98	0.352
Yes	13	4	4	5		
No	92	11	35	46		
AFP level (ng/mL)					2.36	0.351
< 400	69	10	29	30		
≥ 400	36	5	10	21		
TNM stage					2.71	0.243
I–II	95	15	33	47		
III–IV	10	0	6	4		

*HBV* hepatitis B virus, *AFP* alpha-fetoprotein, *TNM* tumor node metastasis

### BCLAF1 expression was positively associated with PD-L1 expression and negatively associated with infiltration levels of immune cells in HCC tissues

**Fig. 2:**
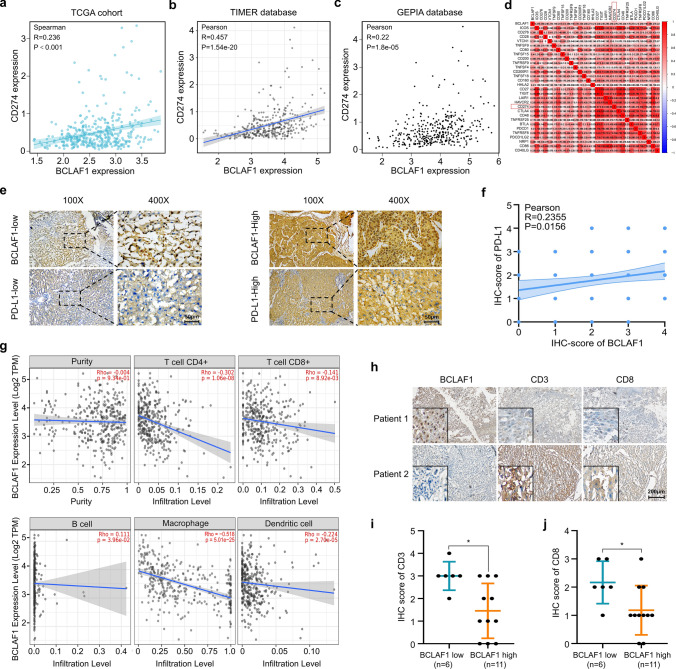
BCLAF1 expression was positively associated with PD-L1 expression and negatively associated with infiltration levels of immune cells in HCC tissues. **a** Correlation analysis between mRNA expression levels of BCLAF1 and PD-L1 in HCC based on the TCGA cohort using R software. **b**, **c** Correlation analysis between mRNA expression levels of BCLAF1 and PD-L1 in HCC based on the TCGA cohort using TIMER (**b**) and GEPIA (**c**) database. **d** Correlation analysis between mRNA expression levels of BCLAF1 and immune checkpoint-related gene expression in HCC based on the TCGA cohort using R software. **e** IHC demonstrated representative images of the different expression levels of BCLAF1 and PD-L1 in paraffin-embedded HCC tissues. Scale bar, 50 μm. **f** Correlation analysis between expression of BCLAF1 and PD-L1 in (**e**) (*n* = 105). **g** Correlation analysis between mRNA expression levels of BCLAF1 and immune cell infiltration levels in HCC using TIMER database.** h** IHC demonstrated representative images of the expression of BCLAF1, CD3 and CD8 in paraffin-embedded HCC tissues. Scale bar, 200 μm. **i, j** Statistical graph of CD3 and CD8 expression in paraffin-embedded HCC tissues based on low (*n* = 6) and high (*n* = 11) expression levels of BCLAF1 in (**h**). All data are shown as mean ± SD (*n* = 3). *P < 0.05

A previous study showed that BCLAF1 upregulates PD-L1 protein levels in breast, prostate, and cervical cancer cell lines in the context of ionizing radiation (IR), and this molecular event was validated by IHC in human esophageal squamous carcinoma tissue [41]. To explore the correlation between BCLAF1 and PD-L1 expression in HCC, we performed a correlation analysis of BCLAF1 and PD-L1 expression levels derived from the TCGA database. The results indicated that the mRNA expression levels of BCLAF1 were positively correlated with PD-L1 (CD274) (Fig. 2a), and consistent findings were obtained using Tumor Immune Estimation Resource (TIMER) database (Fig. 2b) and Gene Expression Profiling Interactive Analysis (GEPIA) database (Fig. 2c) based on TCGA analysis. Furthermore, we analyzed the correlation between BCLAF1 and immune checkpoint-related gene expression, and found that BCLAF1 was positively associated with the expression of immune checkpoint genes, such as *PD-L1* and cytotoxic T lymphocyte-associated antigen-4 (*CTLA4*) (Fig. 2d). To validate the results of TCGA analysis, we used IHC to assess the protein expression of BCLAF1 and PD-L1 in our paraffin-embedded HCC tissues (105 samples). The results showed that the protein expression of BCLAF1 and PD-L1 in HCC was positively correlated (Fig. 2e, f). In addition, to explore the role of BCLAF1 in the immune microenvironment of HCC, the TIMER database was applied to investigate the relationship between BCLAF1 expression and immune cell infiltration. As shown in (Fig. 2g), the mRNA expression levels of BCLAF1 were negatively correlated with the infiltration levels of multiple immune cells (e.g., CD4 + T cells, CD8 + T cells, B cells, macrophage, and dendritic cells) in HCC tissues. Furthermore, we also examined the biomarkers for T‐cell activation (including CD3 and CD8) as well as the protein expression of BCLAF1 via IHC in our collection of paraffin-embedded HCC tissues (50 samples). And the results indicated that BCLAF1 protein expression was negatively correlated with CD3 and CD8 protein expression (Fig. 2h–j). All of these results indicate that BCLAF1 may act as a negative regulator involved in the T cell immune responses of HCC.

### BCLAF1 stabilizes PD-L1 by inhibiting degradation of PD-L1 via the proteasome pathway

**Fig. 3:**
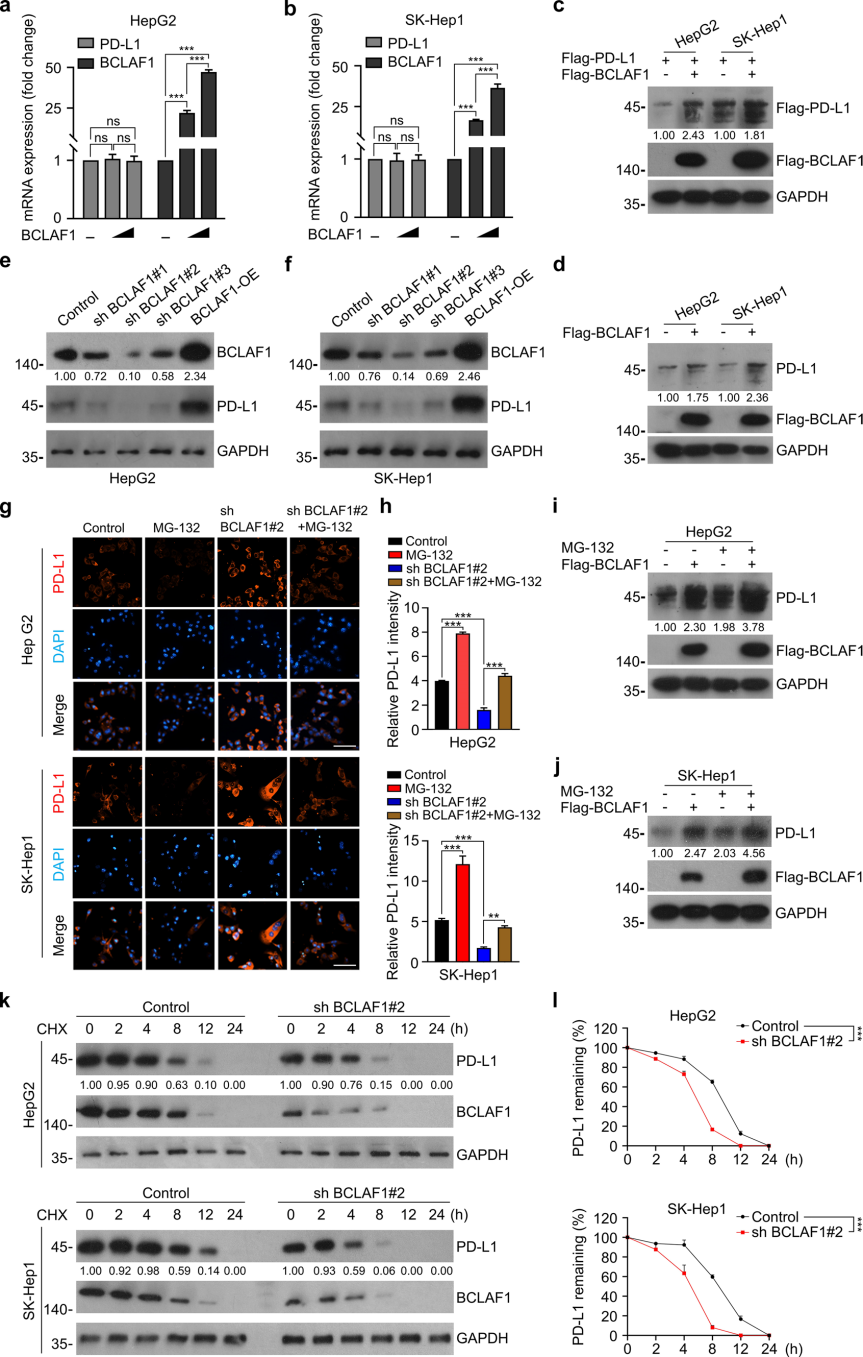
BCLAF1 upregulates PD-L1 expression by inhibiting its proteasome-mediated degradation. **a**, **b** HepG2 (**a**) and SK-Hep1 (**b**) cells were transfected with a concentration gradient of BCLAF1 overexpression plasmid, followed by the qRT-PCR measurement of *PD-L1* mRNA expression levels.** c** Western blot was performed in HepG2 cells and SK-Hep1 cells after co-transfection with Flag-BCLAF1 and Flag-PD-L1 plasmids, respectively. All quantitation was normalized to the protein levels of GAPDH in Control group. **d** Western blot was performed in HepG2 cells and SK-Hep1 cells after transfection with Flag-BCLAF1, respectively. All quantitation was normalized to the protein levels of GAPDH in control group.** e**, **f** HepG2 cells (**e**) and SK-Hep1 cells (**f**) were infected with lentiviruses expressing BCLAF1-specific sh RNA (sh BCLAF1#1, #2, #3), BCLAF1-specific overexpression (BCLAF1-OE), or a control (Control) of the same vector system for 72 h. After infection, whole cell lysates (WCLs) were prepared and BCLAF1 and PD-L1 protein levels were determined by Western blot. All quantitation was normalized to the protein levels of GAPDH in Control group. **g** HepG2 and SK-Hep1 cells with or without BCLAF1 knockdown were treated with or without MG-132 (20 µM) for 8 h before harvesting. Cell immunofluorescence was performed to detect PD-L1 expression. **h** Quantification of Cell immunofluorescence in **g**. Scale bar, 200 μm. **i**, **j** Western blot were performed in HepG2 cells (**i**) and SK-Hep1 cells (**j**) transfected with Flag-BCLAF1 plasmid with or without MG-132 (20 µM) treatment for 8 h before harvesting, respectively. All quantitation was normalized to the protein levels of GAPDH in Control group. **k** The half-life of PD-L1 protein was detected using Western blot on HepG2 and SK-Hep1 cells with stable knockdown of BCLAF1 and Control group by treating them with cycloheximide (CHX) (50 µg/mL) and harvesting the WCLs at different time points. All quantitation was normalized to the protein levels of GAPDH in Control group. **l** Statistics of PD-L1 protein half-life in **k**. All data are shown as mean ± SD (*n* = 3). **P < 0.01, ***P < 0.001

To gain insights into the role of BCLAF1 in the regulation of PD-L1 expression in HCC, we upregulated BCLAF1 expression by transient plasmid transfection in HCC cell lines including HepG2 and SK-Hep1. PD-L1 expression was detected by Quantitative Real-time reverse transcription PCR (qRT-PCR) and Western blot. The results showed that BCLAF1 did not alter the mRNA expression levels of PD-L1 (Fig. 3a, b), but led to increased protein expression of PD-L1 at both exogenous and endogenous levels (Fig. 3c, d), which implied that BCLAF1 modulated the expression of PD-L1 at the post-transcription levels. Additionally, we selected HepG2 and SK-Hep1 cells to construct stable cell lines with BCLAF1 knockdown (Control/sh BCLAF1#1/sh BCLAF1#2/sh BCLAF1#3) and BCLAF1 overexpression (Control/BCLAF1-OE) through lentiviral infection, and the efficiency was validated by Western blot. The results indicated that sh BCLAF1#2 was more effective in knockdown of BCLAF1 in two HCC cell lines compared to sh BCLAF1#1 and sh BCLAF1#3. Also, BCLAF1 was efficiently overexpressed in two HCC cell lines, and thus sh BCLAF2#2 and BCLAF1-OE HCC cell lines were used for subsequent studies (Fig. 3e, f). With stable knockdown or overexpression of BCLAF1, the protein expression of PD-L1 was correspondingly downregulated or upregulated (Fig. 3e, f). One related study revealed that BCLAF1 affects post-translational modifications to stabilize PD-L1 protein in the context of IR in breast, prostate, and cervical cancer cell lines [41]. We speculated that BCLAF1 may affect post-translational modifications of PD-L1 protein in HCC cells. We found that the upregulation of PD-L1 caused by ectopic expression of BCLAF1 could be enhanced through the proteasome inhibitor MG-132 in HepG2 and SK-Hep1 cells, while the downregulation of PD-L1 caused by silencing BCLAF1 was rescued via MG-132 treatment (Fig. 3g–j and Supplemental Fig. 1a, b). Subsequently, cycloheximide (CHX), an inhibitor of protein synthesis, was applied to detect whether BCLAF1 affected the stability of endogenous PD-L1. As shown in Fig. 3k, l, the knockdown of BCLAF1 markedly accelerated the decreased expression of PD-L1. Overall, these results suggest that BCLAF1 stabilizes PD-L1 by inhibiting degradation of PD-L1 via the proteasome pathway.

### BCLAF1 stabilizes PD-L1 via suppressing SPOP-mediated ubiquitination and degradation of PD-L1

**Fig. 4:**
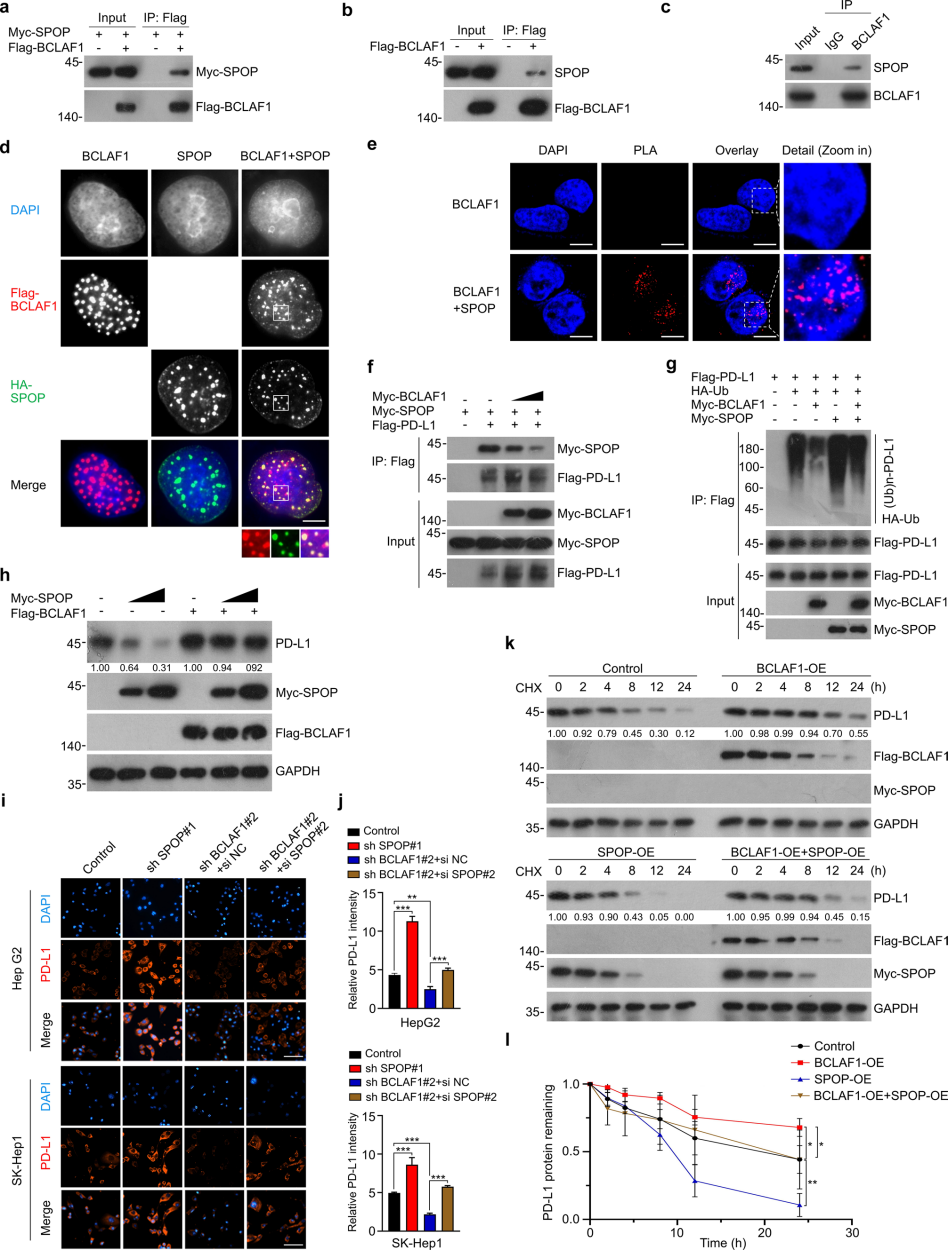
BCLAF1 stabilizes PD-L1 via suppressing SPOP-mediated ubiquitination and degradation of PD-L1. **a** Western blot of WCLs and Co-IP samples harvested from HEK-293 T cells co-transfected with Flag-BCLAF1 and Myc-SPOP plasmids for 24 h. **b** Western blot of WCLs and Co-IP samples harvested from HEK-293 T cells transfected with Flag-BCLAF1 plasmid for 24 h. **c** Western blot of the indicated proteins in WCLs and Co-IP samples of IgG or anti-BCLAF1 antibody obtained from the cell extracts of SK-Hep1 cells. **d** Representative Cell immunofluorescence images of SK-Hep1 cells transfected with Flag-BCLAF1 and/or HA-SPOP plasmids, stained with anti-Flag, anti-HA antibodies and DAPI. Scale bar, 15 μm. **e** Proximity ligation assay (PLA) indicating the interaction of BCLAF1 and SPOP in SK-Hep1 cells (red: PLA positive signal; blue: DAPI, scale bar, 35 μm). **f** HEK-293 T cells were treated with MG-132 (20 μM) for 8 h prior to lysis, and Co-IP analysis was performed to detect the effect of BCLAF1 on the interaction of SPOP with PD-L1 using the antibodies shown. **g** HEK-293 T cells were treated with MG-132 (20 μM) for 8 h prior to lysis, and in vivo ubiquitination assays were performed using the antibodies shown to detect the effect of BCLAF1 on ubiquitination of PD-L1 mediated by SPOP. **h** Western blot was performed on SK-Hep1 cells transfected with a gradient of Flag-BCLAF1 and/or Myc-SPOP plasmids for 24 h. All quantitation was normalized to the protein levels of GAPDH in control group. **i** Representative Cell immunofluorescence images of BCLAF1 knockdown or Control group HepG2 cells and SK-Hep1 cells transfected or not transfected with si SPOP#2 for detection of PD-L1 protein expression after staining with BCLAF1 antibody and DAPI. Scale bar, 200 μm. **j** Quantification of Cell immunofluorescence in **i**. **k** SK-Hep1 cells transfected with Flag-BCLAF1 and/or Myc-SPOP plasmids were treated with CHX (50 µg/mL) and WCLs were harvested at different time points, and the half-life of PD-L1 proteins was detected by Western blot. All quantitation was normalized to the protein levels of GAPDH in control group. **l** Statistics of PD-L1 protein half-life in **k**. All data are shown as mean ± SD (*n* = 3). *P < 0.05, **P < 0.01, ***P < 0.001

To further investigate the mechanism by which BCLAF1 stabilizes PD-L1, co-immunoprecipitation (Co-IP) and liquid chromatography–tandem mass spectrometry (LC–MS/MS) were performed to characterize the underlying substrate proteins of BCLAF1 following its exogenous expression and immunoprecipitation from HEK-293T cells (Table 2). Zhang et al. [12] demonstrated that the E3 ubiquitin ligase SPOP specifically recognizes and mediates ubiquitination and degradation of PD-L1. Therefore, we paid special attention to SPOP in immunoprecipitation complexes of BCLAF1. We first verified the binding of BCLAF1 to SPOP by Co-IP assay. Ectopically expressed Myc-SPOP protein co-precipitated with Flag-BCLAF1 (Fig. 4a); conversely, Myc-BCLAF1 protein co-precipitated with Flag-SPOP (Supplemental Fig. 1c). In HCC cells (SK-Hep1), Flag-BCLAF1 was able to immunoprecipitate endogenous SPOP (Fig. 4b). Importantly, endogenous BCLAF1–SPOP complexes were present in SK-Hep1 cells, as evidenced by Co-IP analysis with BCLAF1 antibody (Fig. 4c). In addition to Co-IP analysis, cell immunofluorescence assay showed that ectopically expressed Flag-BCLAF1 co-localized with HA-SPOP in the nucleus of SK-Hep1 cells (Fig. 4d). To determine whether the interaction between BCLAF1 and SPOP is direct, we used proximity ligation assay (PLA). This assay produces a positive signal only when the distance between the two proteins is less than 40 nm and is highly suited for the direct detection of protein interactions [42]. As shown in Fig. 4e, the PLA results indicated that the experimental group (BCLAF1 primary antibody + SPOP primary antibody) exhibited a significant positive fluorescence signal compared to the negative control group (BCLAF1 primary antibody only). SPOP contains three structural domains: a substrate-binding MATH domain at the N-terminus, a CUL3-binding BTB domain, and a nuclear localization sequence (NLS) domain at the C-terminus [43] (Supplemental Fig. 2a). To determine which domain may mediate its interaction with BCLAF1, we generated three deletion mutants of SPOP (SPOP-ΔMATH, ΔBTB, and ΔNLS) corresponding to the deletion of these three domains, respectively (Supplemental Fig. 2b). Co-IP assay was performed to examine the binding of BCLAF1 with the full-length SPOP (SPOP-WT) and the three deletion mutants. As shown in Supplemental Fig. 2c, SPOP-WT, SPOP-ΔBTB, and SPOP-ΔNLS, but not SPOP-ΔMATH interacted with BCLAF1. Together, these findings indicate a direct interaction between BCLAF1 and SPOP, with the MATH domain of SPOP playing a critical role in the BCLAF1-SPOP interaction.

**Table 2:** The number of total/unique peptides identified by mass spectrometry analysis

Group	Protein name	Flag-BCLAF1 peptide count	Unique peptide count
Novel	SPOP	3	3
	ALDH2	5	5
	LMNB1	19	18
Known	HSP90AA1	35	20
	HSP90AB1	34	17
	CASP14	10	10

Given that SPOP acts as an E3 ubiquitin ligase to mediate ubiquitination and/or degradation of substrates [44]. We first explored whether SPOP affected the protein expression levels and ubiquitination levels of BCLAF1. As shown in Supplemental Fig. 2d, e, SPOP-WT, SPOP-ΔMATH, SPOP-ΔBTB, and SPOP-ΔNLS did not affect the protein expression levels of BCLAF1 in SK-Hep1 cells, and SPOP-WT failed to upregulate the ubiquitination levels of BCLAF1 in HEK-293T cells. In turn, we assessed the effect of BCLAF1 on mRNA and protein expression levels of SPOP. The results showed that overexpression or knockdown of BCLAF1 did not affect the mRNA and protein expression levels of SPOP in SK-Hep1 cells (Supplemental Fig. 2f, g). To this point, we hypothesized that BCLAF1 may stabilize PD-L1 by affecting the ubiquitination and degradation of PD-L1 by SPOP. To verify this hypothesis, we performed Co-IP and in vivo ubiquitination experiments in HEK-293T cells. As depicted in Fig. 4f, g, BCLAF1 inhibited the interaction between SPOP and PD-L1, thereby decreasing the SPOP-mediated ubiquitination level of exogenously expressed Flag-PD-L1. Similarly, BCLAF1 decreased the SPOP-mediated ubiquitination level of endogenous PD-L1 in SK-Hep1 cells (Supplemental Fig. 2h). Notably, we examined the ubiquitination degree of PD-L1 in HCC tissues and found that it was lower in tissues with relatively higher BCLAF1 expression, compared to those with lower BCLAF1 expression (Supplemental Fig. 2i). Moreover, Western blot showed that BCLAF1 overexpression abolished SPOP overexpression-induced downregulation of PD-L1 in SK-Hep1 cells (Fig. 4h). Besides, we selected HepG2 and SK-Hep1 cells to construct stable cell lines with SPOP knockdown (Control/sh SPOP#1/sh SPOP#2/sh SPOP#3) and SPOP overexpression (Control/SPOP-OE) by lentiviral infection. Meanwhile, we designed three specific targeting small interfering RNAs (si RNAs) (si SPOP#1/si SPOP#2/si SPOP#3) for transient silencing of SPOP. All of the above were verified for their efficiency by Western blot, and as shown in Supplemental Fig. 2j, k, sh SPOP#1 and si SPOP#2 showed a more effective gene silencing efficiency, and SPOP-OE was also successful for the SPOP overexpression. Next, cell immunofluorescence was used to assess the role of SPOP in stabilizing PD-L1 by BCLAF1, and the results showed that the protein expression levels of PD-L1 were down-regulated after BCLAF1 knockdown, while SPOP knockdown reversed the down-regulation of PD-L1 protein expression levels induced by BCLAF1 knockdown in HepG2 and SK-Hep1 cells (Fig. 4i, j and Supplemental Fig. 2l, m). Subsequently, CHX was applied to detect the effects of SPOP and BCLAF1 on the stability of endogenous PD-L1. As shown in Fig. 4k, l, BCLAF1 transient overexpression retarded PD-L1 degradation, and SPOP transient overexpression eliminated the retarding effect of BCLAF1 overexpression on PD-L1 protein degradation in SK-Hep1 cells. Together, these results suggest that BCLAF1 stabilizes PD-L1 by inhibiting the ubiquitination and degradation of PD-L1 by SPOP.

### SPOP-binding consensus (SBC) motif-associated BCLAF1 mutant is defective in BCLAF1-SPOP interaction and regulation of the SPOP-PD-L1 axis by BCLAF1

**Fig. 5:**
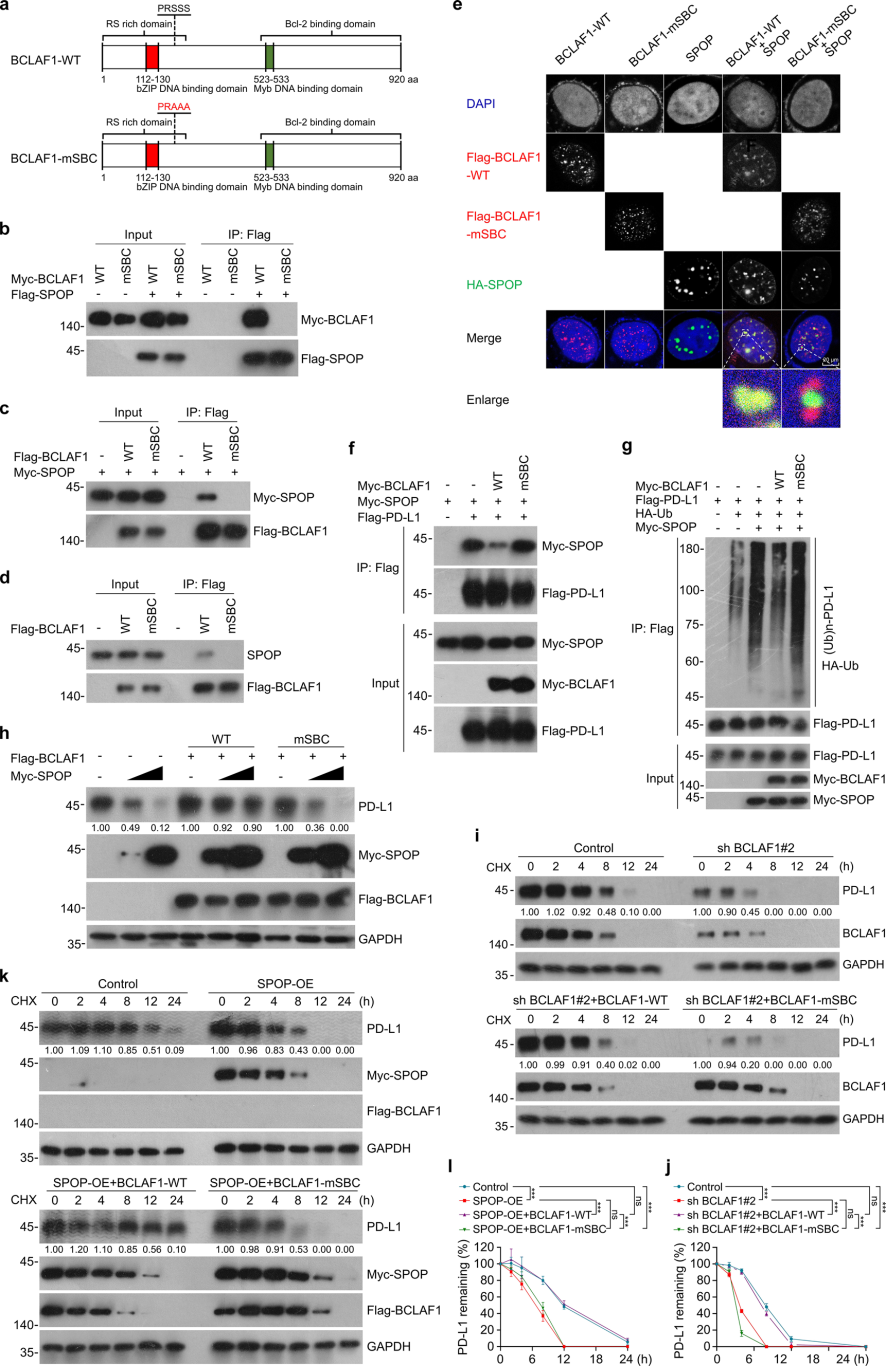
SPOP-binding consensus (SBC) motif-associated BCLAF1 mutant is defective in BCLAF1-SPOP interaction and regulation of the SPOP-PD-L1 axis by BCLAF1. **a** Diagram showing BCLAF1-WT and BCLAF1-SBC motif mutant.** b** Myc-BCLAF1-WT or Myc-BCLAF1-mSBC plasmids were co-transfected with Flag-SPOP plasmid in HEK-293 T cells for 24 h. Harvested WCLs and Co-IP samples were analyzed by Western blot. **c** Flag-BCLAF1-WT or Flag-BCLAF1-mSBC plasmids were co-transfected with Myc-SPOP plasmid in HEK-293 T cells for 24 h. Harvested WCLs and Co-IP samples were analyzed by Western blot.** d** Flag-BCLAF1-WT or Flag-BCLAF1-mSBC plasmids were transfected in HEK-293 T cells for 24 h. Harvested WCLs and Co-IP samples were analyzed by Western blot. **e** Representative Cell immunofluorescence images of SK-Hep1 cells transfected with Flag-BCLAF1-WT, Flag-BCLAF1-mSBC and HA-SPOP plasmids, stained with anti-Flag, anti-HA antibodies and DAPI. Scale bar, 20 μm. **f** HEK-293 T cells were treated with MG-132 (20 μM) for 8 h prior to lysis, and Co-IP analysis was performed to detect the effect of BCLAF1-WT and BCLAF1-mSBC on the interaction of SPOP with PD-L1 using the antibodies shown.** g** HEK-293 T cells were treated with MG-132 (20 μM) for 8 h prior to lysis, and in vivo ubiquitination assays were performed using the antibodies shown to detect the effect of BCLAF1-WT and BCLAF1-mSBC on ubiquitination of PD-L1 mediated by SPOP. **h** Western blot was performed on SK-Hep1 cells transfected with a gradient of Flag-BCLAF1-WT, Flag-BCLAF1-mSBC and/or Myc-SPOP plasmids for 24 h. All quantitation was normalized to the protein levels of GAPDH in Control group. **i** SK-Hep1 cells with knockdown of BCLAF1 were transfected with Flag-BCLAF1-WT or Flag-BCLAF1-mSBC plasmids, and WCLs were harvested at different time points and the half-life of the PD-L1 protein was detected by Western blot. All quantitation was normalized to the protein levels of GAPDH in control group. **j** Statistics of PD-L1 protein half-life in **i**. **k** SK-Hep1 cells with overexpression of SPOP were transfected with Flag-BCLAF1-WT or Flag-BCLAF1-mSBC plasmids, and WCLs were harvested at different time points and the half-life of the PD-L1 protein was detected by Western blot. All quantitation was normalized to the protein levels of GAPDH in control group.** l** Statistics of PD-L1 protein half-life in **k**. All data are shown as mean ± SD (n = 3). ***P < 0.001, ns *P* ≥ 0.05

Previous studies reported that one or more SBC motifs (θ-π-S–S/T-S/T; θ: nonpolar residues, π: polar residues) are present in known SPOP substrates [45–47]. We carried out a protein motif search on BCLAF1 sequence and found only one excellently matched SBC motif (137- “PRSSS”-141 aa). To check whether this potential motif is required for BCLAF1-SPOP interaction, we generated a BCLAF1-mSBC mutant (BCLAF1-mSBC) in which the third to fifth Ser residues in the putative motif sequence were replaced by the nonpolar amino acid, Alanine (137- “PRAAA”-141 aa) (Fig. 5a). Co-IP analysis and GST pull-down assay showed that BCLAF1-mSBC completely eliminated the BCLAF1-SPOP interaction at exogenous or semi-endogenous levels (Fig. 5b–d and Supplemental Fig. 3a). Cell immunofluorescence indicated that BCLAF1-mSBC strongly disrupted the co-localization of BCLAF1 with SPOP in SK-Hep1 cells (Fig. 5e). Next, we further investigated the effect of BCLAF1-mSBC on SPOP-PD-L1 interaction as well as SPOP-mediated ubiquitination of PD-L1. Co-IP analysis revealed that BCLAF1-mSBC eliminated the competitive inhibitory effect of BCLAF1 on SPOP-PD-L1 interaction (Fig. 5f). In vivo ubiquitination analysis suggested that BCLAF1-mSBC eradicated the inhibitory effect of BCLAF1 on SPOP-mediated ubiquitination of exogenous PD-L1 (Fig. 5g). Besides, BCLAF1-mSBC also nullified the inhibitory effect of BCLAF1 on SPOP-mediated ubiquitination of endogenous PD-L1 (Supplemental Fig. 3b). Finally, we checked the effect of BCLAF1-mSBC on the stabilization of PD-L1 expression levels by BCLAF1. Western blot showed that BCLAF1-mSBC disrupted the inhibitory effect of BCLAF1 on SPOP-mediated down-regulation of PD-L1 expression levels (Fig. 5h). Furthermore, in BCLAF1-silenced SK-Hep1 cells, we performed exogenous overexpression of BCLAF1 wild-type (BCLAF1-WT) and BCLAF1-mSBC, respectively, to detect the half-life of endogenous PD-L1. The results showed that overexpression of BCLAF1-WT significantly prolonged the half-life of PD-L1, whereas BCLAF1-mSBC lost this effect (Fig. 5i, j). Similarly, in SPOP overexpressed SK-Hep1 cells, we performed exogenous overexpression of BCLAF1-WT and BCLAF1-mSBC, respectively, and examined the change in half-life of endogenous PD-L1. The results indicated that overexpression of BCLAF1-WT eliminated the accelerating effect of SPOP overexpression on the half-life of PD-L1, whereas BCLAF1-mSBC failed (Fig. 5k, l). Collectively, we have identified an SBC motif present in BCLAF1 that is essential for the BCLAF1-SPOP interaction and the regulatory role of BCLAF1 on the SPOP-PD-L1 axis.

### BCLAF1 induces immune escape of HCC cells partly in an SPOP-PD-L1 axis-dependent manner in vitro

Current evidence supports the role of BCLAF1 as an oncogenic protein in HCC [22–24], whereas SPOP has been reported as an tumor suppressor in HCC [18–20]. Consistent findings were also obtained in our study. Indeed, knockdown of BCLAF1 (Supplemental Fig. 4a) inhibited proliferation (Supplemental Fig. 4b–f), migration (Supplemental Fig. 4g–l), and invasion (Supplemental Fig. 4m–o) of HepG2 and SK-Hep1 cells compared with Control group, whereas the opposite result was observed with BCLAF1 overexpression (Supplemental Fig. 4). Furthermore, knockdown of SPOP (Supplemental Fig. 5a) significantly reversed the promotional impact of BCLAF1 on HCC cell proliferation (Supplemental Fig. 5b–f), migration (Supplemental Fig. 5g–l), and invasion (Supplemental Fig. 5m–o), suggesting that BCLAF1 is partially dependent on SPOP to promote the malignant phenotype of HCC.

However, the function of BCLAF1 in the tumor immune microenvironment is still unclear. To investigate the potential role of BCLAF1 in tumor immunity of HCC, an in vitro co-culture model of Jurkat cells and HCC cells was established. Specifically, we used the HCC cell lines with the control group, BCLAF1 stable overexpression (BCLAF1-OE), and BCLAF1 stable knockdown (sh BCLAF1#2). Besides, transient transfection of si NC (sh BCLAF1#2 + si NC) and si SPOP#2 (sh BCLAF1#2 + si SPOP#2) was performed in sh BCLAF1#2 HCC cells for SPOP knockdown. Subsequently, HCC cells were co-cultured with Jurkat cells for 24 h after treatment with DMSO or Atezolizumab, respectively. The Jurkat cell line is an immortalized T lymphocyte cell line and is most often used as a prototypical T cell line for the study of T cell signaling [41]. Atezolizumab, a selective, high-affinity monoclonal antibody to human IgG, specifically block the binding of PD-L1 to PD-1, restoring the immune surveillance function of T cells, which in turn helps T cells recognize and kill tumor cells, and has been used as a first-line treatment for advanced HCC [3, 7].

**Fig. 6:**
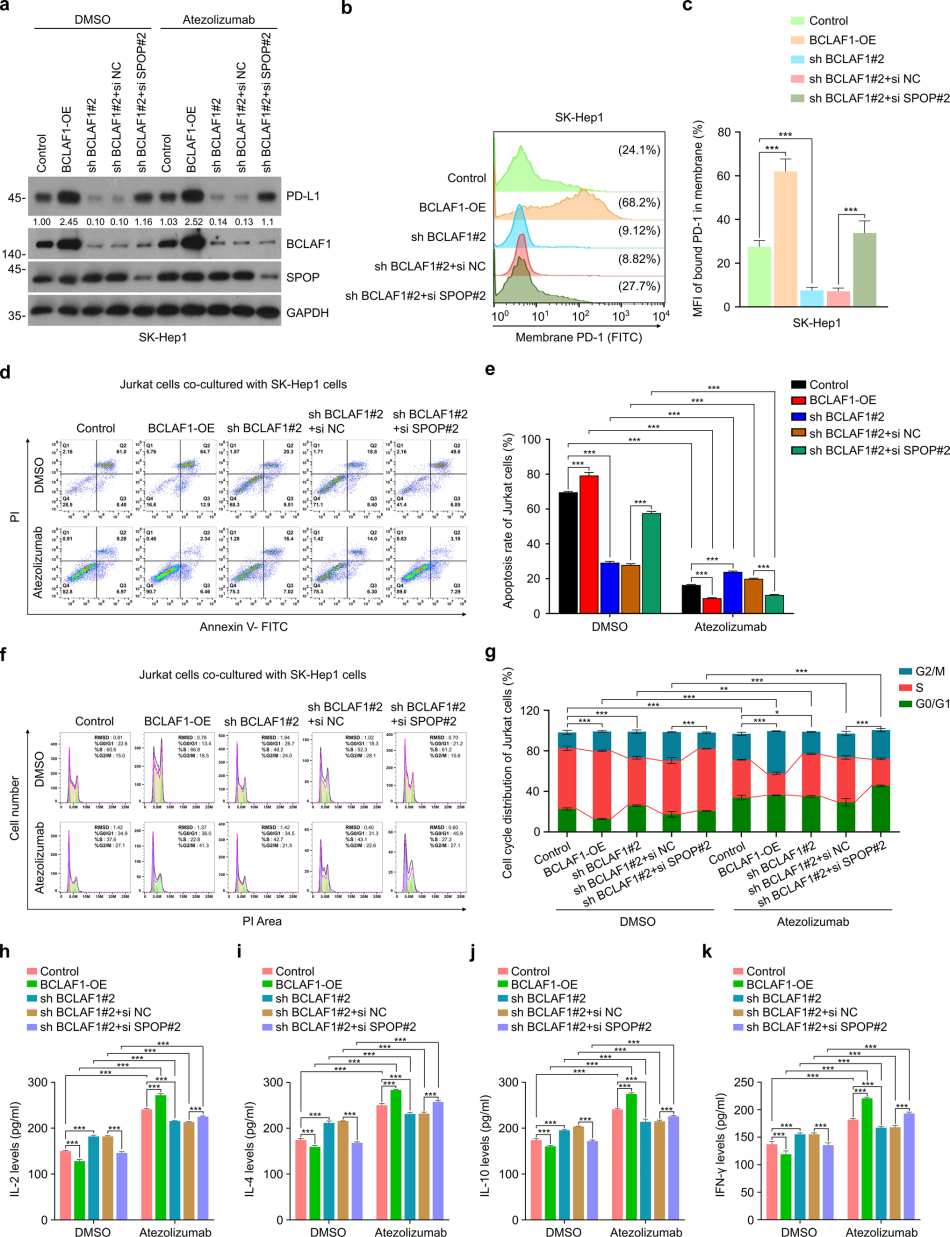
BCLAF1 induces immune escape of HCC cells partly in an SPOP-PD-L1 axis-dependent manner. **a** SK-Hep1 cells achieving BCLAF1 overexpression, BCLAF1 knockdown, and BCLAF1 knockdown followed by SPOP knockdown were co-cultured with Jurkat cells for 24 h after treatment with DMSO or Atezolizumab (10 ng/mL). Western blot of SK-Hep1 cells in the HCC cell-Jurkat cell co-culture system for detection of PD-L1 expression levels. All quantitation was normalized to the protein levels of GAPDH in Control group. **b** SK-Hep1 cells achieving BCLAF1 overexpression, BCLAF1 knockdown, and BCLAF1 knockdown followed by SPOP knockdown were co-cultured with Jurkat cells for 24 h. Flow cytometry analysis of PD-1 binding on SK-Hep1 cell surface to determine the effect of BCLAF1 on PD-1/PD-L1 binding. **c** Statistics of mean fluorescence intensity (MFI) for PD-1 in (**b**). **d** SK-Hep1 cells achieving BCLAF1 overexpression, BCLAF1 knockdown, and BCLAF1 knockdown followed by SPOP knockdown were co-cultured with Jurkat cells for 24 h after treatment with DMSO or Atezolizumab (10 ng/mL). Apoptosis levels of Jurkat cells were detected by Flow cytometry analysis.** e** Statistics of apoptotic levels of Jurkat cells in **d**.** f** SK-Hep1 cells achieving BCLAF1 overexpression, BCLAF1 knockdown, and BCLAF1 knockdown followed by SPOP knockdown were co-cultured with Jurkat cells for 24 h after treatment with DMSO or Atezolizumab (10 ng/mL). Cell cycles of Jurkat cells were detected by flow cytometry analysis.** g** Statistics of cell cycle of Jurkat cells in **f**. **h**–**k** SK-Hep1 cells achieving BCLAF1 overexpression, BCLAF1 knockdown, and BCLAF1 knockdown followed by SPOP knockdown were co-cultured with Jurkat cells for 24 h after treatment with DMSO or Atezolizumab (10 ng/mL). The levels of IL-2 (**h**), IL-4 (**i**), IL-10 (**j**), and IFN-γ (**k**) produced by Jurkat cells were detected by ELISA. All data are shown as mean ± SD (n = 3). *P < 0.05, **P < 0.01, ***P < 0.001

First, Western blot was used to detect the expression levels of PD-L1 in HCC cells. The results showed that overexpression of BCLAF1 increased the expression levels of PD-L1 in HCC cells compared to control group, and knockdown of BCLAF1 exhibited the opposite result; while knockdown of SPOP reversed the down-regulation of the expression levels of PD-L1 induced by knockdown of BCLAF1 (Fig. 6a, Supplemental Fig. 6a). Moreover, there was no significant change in PD-L1 expression levels after the treatment of Atezolizumab compared to the DMSO (Fig. 6a, Supplemental Fig. 6a). To assess the efficacy of Atezolizumab in blocking the binding of PD-L1/PD-1 in the co-culture model, we measured the amount of PD-1 binding to SK-Hep1 cell membranes using the PD-L1/PD-1 binding assay. The results indicated that Atezolizumab almost completely hindered PD-L1/PD-1 binding compared to the DMSO group (Supplemental Fig. 6b, c). Next, we investigated whether BCLAF1-induced PD-L1 upregulation enhances PD-1 binding on HCC cells to determine the functional importance of heightened PD-L1 expression by BCLAF1. The findings demonstrate that heightened expression of BCLAF1 led to increased PD-1 binding to PD-L1 on the membrane of SK-Hep1 cells, whereas silencing BCLAF1 had the opposite effect. Additionally, silencing SPOP counteracted the reduction in PD-1/PD-L1 binding observed upon silencing BCLAF1 (Fig. 6b, c). Further, to determine whether increased PD-1 binding due to BCLAF1-mediated upregulation of PD-L1 affects T cell function, we examined apoptosis and cell cycle of Jurkat cells by Flow cytometry, and the levels of cytokines secreted by Jurkat cells via ELISA. The results indicate that overexpressing BCLAF1 resulted in higher levels of apoptosis (Fig. 6d, e and Supplemental Fig. 6d, e) and a greater proportion of cells in the S phase (Fig. 6f, g and Supplemental Fig. 6f, g). Additionally, there was a decrease in cytokine secretion from Jurkat cells, including Interleukin-2 (IL-2) (Fig. 6h, Supplemental Fig. 6h), IL-4 (Fig. 6i, Supplemental Fig. 6i), IL-10 (Fig. 6j, Supplemental Fig. 6j), and Interferon-γ (IFN-γ) (Fig. 6k, Supplemental Fig. 6k). Conversely, knockdown of BCLAF1 produced the opposite effects, suggesting that BCLAF1 promoted immunosuppression. In addition, knockdown of SPOP effectively reversed the effects of BCLAF1 knockdown on apoptosis, cell cycle, and secreted cytokines of Jurkat cells (Fig. 6d–k, Supplemental Fig. 6b–i), indicating that BCLAF1 partially depended on SPOP to participate in the suppression of the anti-tumor immune microenvironment. We then hypothesized whether BCLAF1 affects the efficacy of ICB therapy, which is important for the introduction of BCLAF1 as a potential therapeutic target for enhanced ICB therapy in HCC. In this regard, we performed co-culture systems with or without anti-PD-L1 monoclonal antibody Atezolizumab. As expected, Atezolizumab decreased the level of apoptosis and the percentage of S-phase cells, and increased the level of cytokines secreted by Jurkat cells compared to the DMSO group (Fig. 6d–k, Supplemental Fig. 6d–k). Moreover, the results indicated that the efficacy of Atezolizumab on apoptosis, cell cycle, and cytokine levels of Jurkat cells was augmented by the overexpression of BCLAF1, while the opposite effect was observed upon *BCLAF1* knockdown (Fig. 6d–k, Supplemental Fig. 6d–k). This demonstrates that BCLAF1 enhances the responsiveness of HCC cells to anti-PD-L1 treatment. Together, these findings suggest that BCLAF1 facilitates the immune evasion of HCC cells through modulation of the SPOP-PD-L1 axis. Additionally, BCLAF1 overexpression reinforces the impact of anti-PD-L1 treatment in an in vitro co-culture system.

### SPOP-binding consensus (SBC) motif-associated BCLAF1 mutant is defective in inducing immune escape of HCC cells

**Fig. 7:**
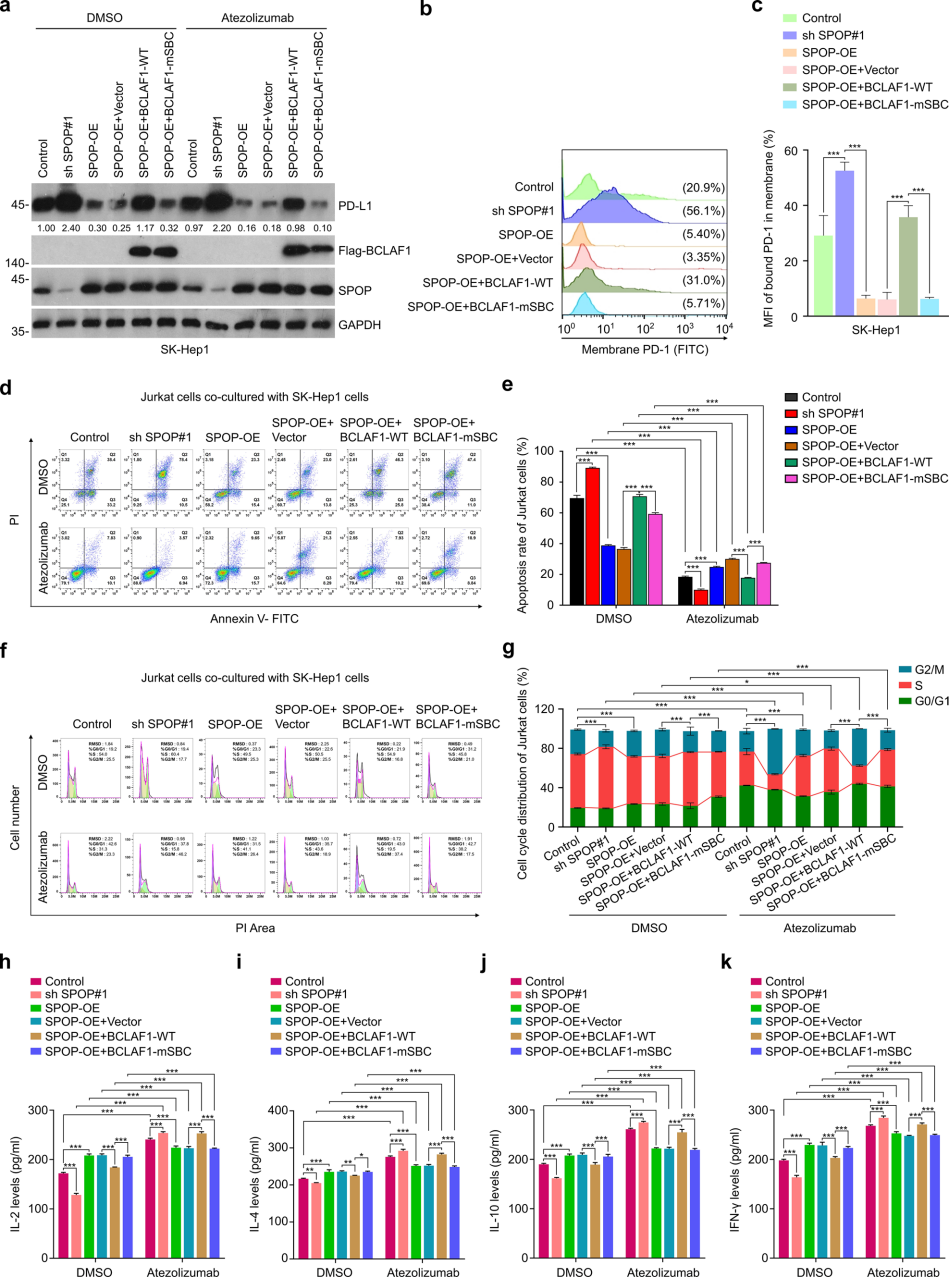
SPOP-binding consensus (SBC) motif-associated BCLAF1 mutant is defective in inducing immune escape of HCC cells. **a** SK-Hep1 cells achieving SPOP knockdown, SPOP overexpression, and exogenous overexpression of BCLAF1-WT and BCLAF1-mSBC after SPOP overexpression were co-cultured with Jurkat cells for 24 h after treatment with DMSO or Atezolizumab (10 ng/mL). Western blot of SK-Hep1 cells in the HCC cell-Jurkat cell co-culture system for detection of PD-L1 expression levels. All quantitation was normalized to the protein levels of GAPDH in Control group. **b** SK-Hep1 cells achieving SPOP knockdown, SPOP overexpression, and exogenous overexpression of BCLAF1-WT and BCLAF1-mSBC after SPOP overexpression were co-cultured with Jurkat cells for 24 h. Flow cytometry analysis of PD-1 binding on SK-Hep1 cell surface to determine the effect of BCLAF1 on PD-1/PD-L1 binding. **c** Statistics of mean fluorescence intensity (MFI) for PD-1 in **b**. **d** SK-Hep1 cells achieving SPOP knockdown, SPOP overexpression, and exogenous overexpression of BCLAF1-WT and BCLAF1-mSBC after SPOP overexpression were co-cultured with Jurkat cells for 24 h after treatment with DMSO or Atezolizumab (10 ng/mL). Apoptosis levels of Jurkat cells were detected by Flow cytometry analysis.** e** Statistics of apoptotic levels of Jurkat cells in **d**.** f** SK-Hep1 cells achieving SPOP knockdown, SPOP overexpression, and exogenous overexpression of BCLAF1-WT and BCLAF1-mSBC after SPOP overexpression were co-cultured with Jurkat cells for 24 h after treatment with DMSO or Atezolizumab (10 ng/mL). Cell cycle of Jurkat cells were detected by Flow cytometry analysis.** g** Statistics of cell cycle of Jurkat cells in **f**.** h**–**k** SK-Hep1 cells achieving SPOP knockdown, SPOP overexpression, and exogenous overexpression of BCLAF1-WT and BCLAF1-mSBC after SPOP overexpression were co-cultured with Jurkat cells for 24 h after treatment with DMSO or Atezolizumab (10 ng/mL). The levels of IL-2 (**h**), IL-4 (**i**), IL-10 (**j**), and IFN-γ (**k**) produced by Jurkat cells were detected by ELISA. All data are shown as mean ± SD (n = 3). *P < 0.05, **P < 0.01, ***P < 0.001

Since mutants of the SBC-associated BCLAF1 display deficiencies in BCLAF1-SPOP interactions and BCLAF1's regulation of the SPOP-PD-L1 axis, we investigated whether BCLAF1-mSBC influences BCLAF1's function in HCC tumor immunity. As expected, HCC cells exhibited a rise in PD-L1 expression when *SPOP* was knocked down, while the inverse outcome was observed in the SPOP-overexpressing group (Fig. 7a, Supplemental Fig. 7a). Furthermore, transient overexpression of BCLAF1-WT or BCLAF1-mSBC was performed in HCC cell lines that stably overexpressed SPOP. Western blot analysis indicated that BCLAF1-WT overexpression reversed the down-regulation of PD-L1 expression levels induced by SPOP overexpression, while BCLAF1-mSBC overexpression did not exhibit this effect (Fig. 7a, Supplemental Fig. 7a). Besides, no significant change in PD-L1 expression levels was observed after Atezolizumab treatment compared to the DMSO group (Fig. 7a, Supplemental Fig. 7a). Next, we investigated the impact of BCLAF1-mSBC on PD-1/PD-L1 binding on SK-Hep1 cell membranes. As shown in Fig. 7b, c, overexpression of SPOP resulted in a decrease in PD-L1 expression levels that led to a reduction in PD-1 binding on the cell membranes. Overexpression of wild-type BCLAF1 reversed this effect, but not the BCLAF1-mSBC (Fig. 7b, c). Finally, we examined the activity and function of Jurkat cells. The findings indicated that knockdown of *SPOP* in the DMSO group resulted in increased apoptosis (Fig. 7d, e and Supplemental Fig. 7b, c) and heightened percentage of S-phase cells (Fig. 7f, g and Supplemental Fig. 7d, e), along with decreased levels of cytokines (IL-2, IL-4, IL-10, and INF-γ) released by Jurkat cells (Fig. 7h–k and Supplemental Fig. 7f–i). Conversely, SPOP overexpression produced opposite results (Fig. 7d–k and Supplemental Fig. 7b–i). Meanwhile, we performed transient overexpression of BCLAF1-WT or BCLAF1-mSBC in SPOP-overexpressing HCC cell lines, respectively, and observed that BCLAF1-WT significantly reversed the modulation of apoptosis, cell cycle, and cytokine levels in Jurkat cells induced by SPOP overexpression, whereas BCLAF1-mSBC group lost these effects (Fig. 7d–k and Supplemental Fig. 7b–i). Additionally, SPOP overexpression in the Atezolizumab group invalidated Atezolizumab's effects on Jurkat cells' function, indicating a lessened therapeutic effect (Fig. 7d–k and Supplemental Fig. 7b–i). Corresponding to the DMSO group, we performed transient overexpression of BCLAF1-WT or BCLAF1-mSBC in SPOP-overexpressing HCC cell lines in the Atezolizumab group, respectively. The findings indicate that BCLAF1-WT eliminated the inhibitory effect of SPOP overexpression on Atezolizumab treatment. However, this effect was not observed in BCLAF-mSBC (Fig. 7d–k and Supplemental Fig. 7b–i). In summary, these results demonstrate that the SBC motif mutation disrupted the effects of BCLAF1-WT in immune escape and ICB treatment sensitivity in HCC in vitro.

## Discussion

The expression levels of PD-L1 in tumor cells possibly are associated with the clinical response and efficacy of anti-PD-1/PD-L1 therapies [48]. Therefore, it is necessary to fully understand the molecular mechanisms that regulate PD-L1 expression. Recent studies have described different mechanism controlling PD-L1 abundance at the post-translational levels, such as SPOP-mediated degradation via the proteasomal pathway [12, 15], but the upstream regulation of ubiquitin–proteasome pathway-associated degradation of PD-L1 is not yet fully understood.

**Fig. 8:**
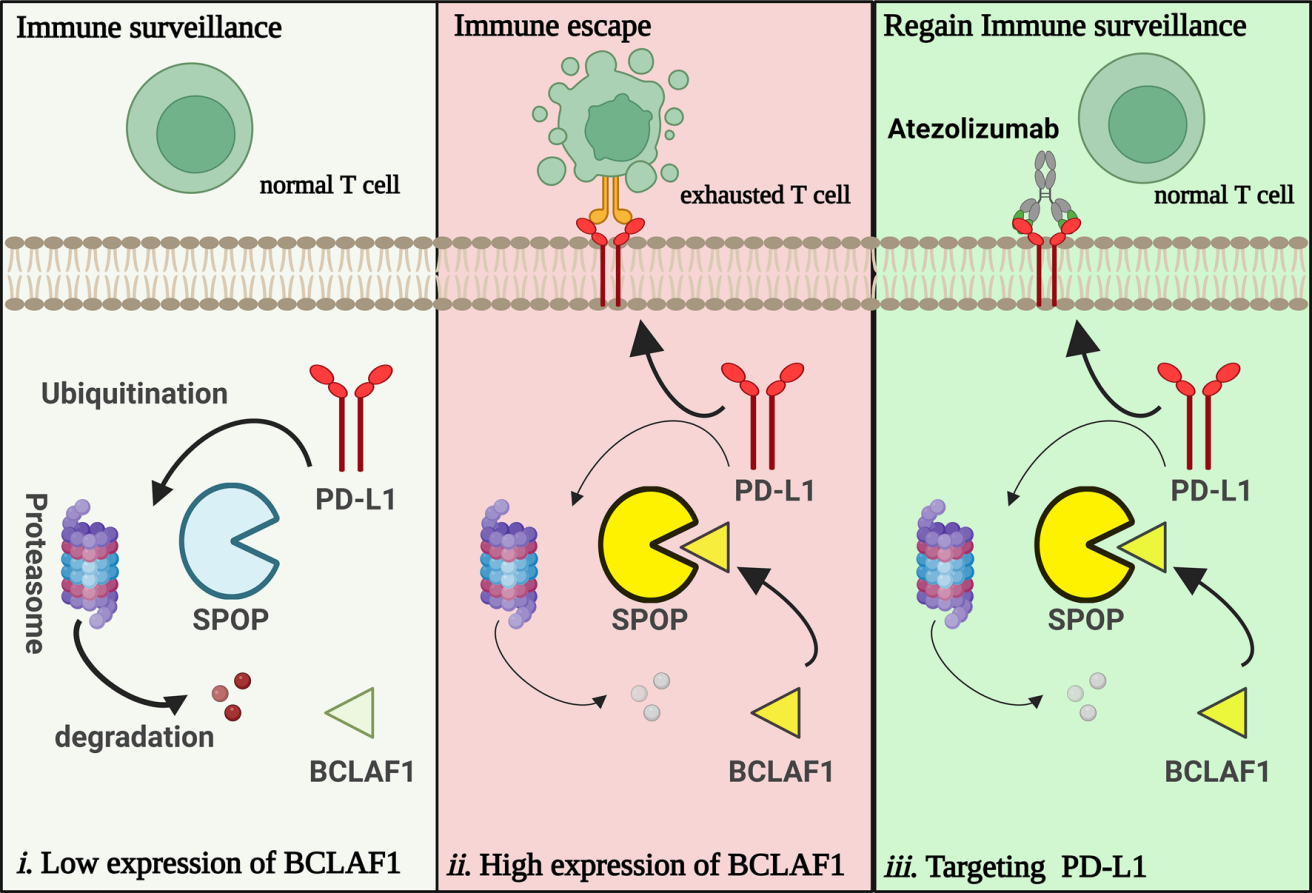
Model diagrams proposed based on the results of this study. **i** Under physiological conditions, BCLAF1 is relatively lowly expressed, SPOP mediates ubiquitination and degradation of PD-L1 to maintain relatively low expression levels of PD-L1, and T cells have normal activity with normal immune surveillance function. **ii** In HCC, BCLAF1 is relatively highly expressed and competitively binds to SPOP to suppress the SPOP-mediated ubiquitination and degradation of PD-L1, thereby releasing more PD-L1 to enhance PD-L1/PD-1 signaling activation, which ultimately leads to T cell exhaustion and immune escape. **iii** Given high expression of BCLAF1 in HCC, the overexpression of PD-L1 is induced by BCLAF1-induced suppression of SPOP-mediated ubiquitination and degradation of PD-L1, treating with Atezolizumab to specifically block the binding of PD-L1 and PD-1 could restore normal T cell activity and immune surveillance function in HCC

Here, we identified BCLAF1 as a novel regulator of PD-L1 in HCC, and it is high expressed in HCC with poor prognosis. Mechanistically, BCLAF1 competitively inhibits SPOP-mediated ubiquitination and degradation of PD-L1 to promote HCC progression and evasion of immune surveillance, and overexpression of BCLAF1 enhances the efficacy of ICB therapy treated with Atezolizumab (Fig. 8). In addition, we identified an SBC motif on BCLAF1 protein, and mutation of this SBC motif disrupted BCLAF1-induced suppression of the SPOP-PD-L1 axis.

A recent study reported that BCLAF1 upregulated PD-L1 expression under IR context in breast cancer, human fibrosarcoma, and prostate cancer cells [41]. Further mechanistic studies have shown that BCLAF1 interacts with PD-L1; moreover, BCLAF1 upregulates the expression of CMTM6, a deubiquitinating enzyme of PD-L1, which in turn inhibits the ubiquitination and degradation of PD-L1 [41], suggesting that BCLAF1 is involved in the regulation of PD-L1 abundance at the post-translational levels. Here, we found that BCLAF1 could upregulate PD-L1 protein levels in a non-IR context in HCC (Fig. 3c, d). Furthermore, we demonstrate that BCLAF1 interacts with SPOP but is not a typical substrate for SPOP because SPOP does not mediate ubiquitination and degradation of BCLAF1 (Supplemental Fig. 2d, e). In turn, BCLAF1 inhibits the interaction between SPOP and PD-L1, thus suppressing the SPOP-mediated ubiquitination of PD-L1 (Fig. 4f, g and Supplemental Fig. 2h). Recently, a series of reports indicated that BCLAF1 drives HCC by positively regulating the HIF-1α pathway [22–24]. For this reason, we also explored whether the interaction of SPOP with BCLAF1 affects the HIF-1α pathway. As shown in (Supplemental Fig. 8a–c), SPOP did not affect the protein and mRNA expression levels of HIF-1α. Together, these results suggest that BCLAF1 may be an upstream regulator of SPOP. Consistent with the results of Ma Z et al*.* [41], we found BCLAF1 in Flag-PD-L1 immunoprecipitates, except for SPOP. Additionally, we observed that upregulation of BCLAF1 inhibited the interaction between SPOP and PD-L1 (Supplemental Fig. 8d). To find the real direct interaction partner of BCLAF1, we performed Co-IP experiments. As shown in Supplemental Fig. 8e, the knockdown of SPOP inhibited the interaction between BCLAF1 and PD-L1, while overexpression of SPOP had the opposite result, suggesting SPOP may partly act as an intermediate between the interaction between BCLAF1 and PD-L1.

Previous studies have found that BCLAF1 is abnormally highly expressed in HCC and that high levels of BCLAF1 are strongly associated with poor prognosis in HCC patients [22–25], as validated by our data (Fig. 1). However, the mechanism of the abnormally high expression of BCLAF1 in HCC has not been reported. Therefore, it is interesting to explore the molecular mechanisms underlying the abnormally high expression of BCLAF1 in HCC. In other tumors, the expression of BCLAF1 is regulated at multiple levels. For example, NF-κB and histone methyltransferase SET and MYND domain-containing protein 3 (SMYD3) were reported to be directly involved in the transcriptional regulation of BCLAF1 in diffuse large B cell lymphoma and bladder cancer, respectively [49]. Splicing factor SRSF10 was reported to be involved in the mRNA splicing of BCLAF1 in colorectal cancer [50]. In addition, it was shown that miR-194-5p was directly involved in the transcriptional regulation of BCLAF1 by binding to BCLAF1 3'UTR, thus inhibiting its transcription or translation in acute granulocytic leukemia and bladder cancer [51–53]. However, in HCC, studies on the regulation of BCLAF1 are relatively limited. At the transcriptional levels, Wen et al*.* [22] showed that HIF-1α activates the transcription of BCLAF1; at the post-translational levels, Zhou et al*.* [25] found that heat shock protein 90α (Hsp90α) binds to BCLAF1 and stabilizes its protein structure, thus preventing the degradation of BCLAF1 via the ubiquitin–proteasome pathway. Given that the upstream regulation of BCLAF1 in HCC remains unclear, we analyzed and predicted the potential regulatory molecules of BCLAF1 in HCC. For instance, at the transcriptional levels, we analyzed the GRTD, HumanTFDB, and PROMO databases and predicted ten shared transcription factors (Supplemental Fig. 9a, Supplemental Table 2). As for the post-transcriptional levels, we took advantage of the miRDB, mirDIP, miRWalk, and TargetScan databases and inferred five shared miRNAs (Supplemental Fig. 9b, Supplemental Table 3). At the post-translational levels, we predicted twenty potential E3 ubiquitin ligases targeting BCLAF1 using the ubibrowser 1.0 database (Supplemental Fig. 9c, Supplemental Table 4). These predicted transcription factors, miRNAs, and E3 ligases need to be further validated in future, such as using chromatin immunoprecipitation techniques (ChIP), dual luciferase reporter assays, in vivo ubiquitination experiments, Co-IP, etc.

In conclusion, we identified BCLAF1 as an upstream regulator of SPOP. It is conceivable that BCLAF1 binding to SPOP may prevent substrate binding or cause conformational changes of SPOP, thereby interfering with or preventing ubiquitin transfer to PD-L1. Thus, BCLAF1 enhances PD-L1 stability and expression through SPOP functional inactivation, resulting in cancer cells evading immune surveillance. Furthermore, BCLAF1 overexpression-mediated stabilization of PD-L1 expression significantly increased the efficacy of ICB therapy. However, the mutations in the SBC motif disrupted the ability of BCLAF1-WT to regulate tumor immunity providing BCLAF1 and its SBC motif an attractive target for designing antitumor therapies in HCC.

### Supplementary Information

Below is the link to the electronic supplementary material.

Supplementary file1 (PDF 1993 KB)

Supplementary file2 (PDF 1987 KB)

## Data Availability

All data generated or analyzed during this study are included in this published article.
