# Supplementary material for: BCLAF1 binds SPOP to stabilize PD-L1 and promotes the development and immune escape of hepatocellular carcinoma
Source: Cell Mol Life Sci. 2024 Feb 10;81(1):82. doi: 10.1007/s00018-024-05144-z (PMC10858942; doi:10.1007/s00018-024-05144-z)
Supplement: Supplementary file 2 — Supplementary file2 (PDF 1987 KB) [file 18_2024_5144_MOESM2_ESM.pdf]

Supplementary Materials

Title: BCLAF1 binds SPOP to stabilize PD-L1 and promote the development and immune escape of hepatocellular carcinoma

Authors:

Zongdong Yu, Xiang Wu, Jie Zhu, Huan Yan, Yuxuan Li, Hui Zhang, Yeling Zhong, Man Lin, Ganghui Ye, Xinming Li, Jiabei Jin, Kailang Li, Jie Wang, Hui Zhuang, Ting Lin, Jian He, Changjiang Lu, Zeping Xu, Xie Zhang, Hong Li, Xiaofeng Jin

Correspondence:

Hong Li, Email: lancet2017@163.com.

Xiaofeng Jin, Email: jinxiaofeng@nbu.edu.cn.

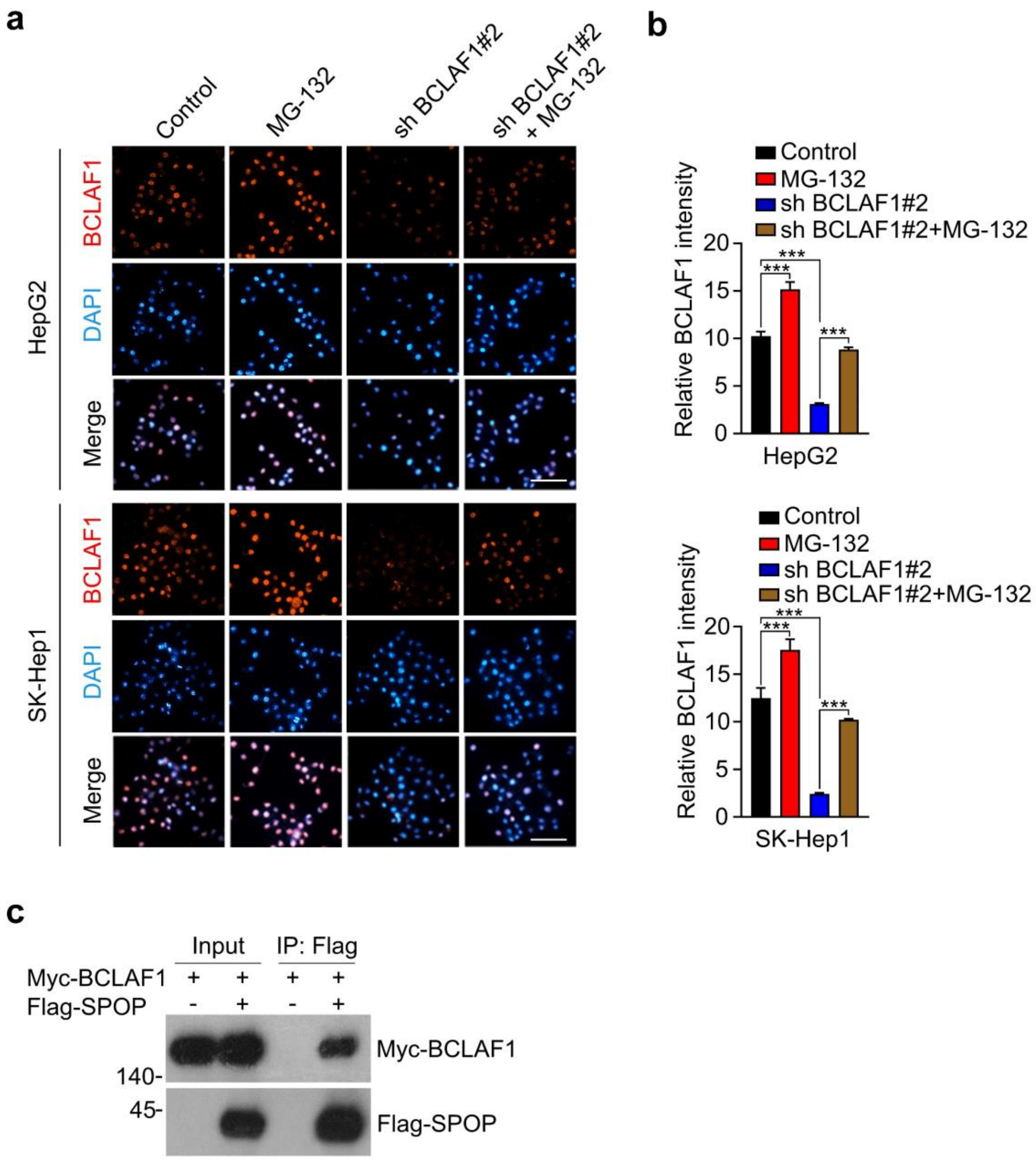

Supplemental Fig. 1 Cellular immunofluorescence for BCLAF1 expression in HCC cells and Co-IP for BCLAF1-SPOP interaction

a Representative images of Cell immunofluorescence for detection of BCLAF1 expression in HepG2 and SK-Hep1 cells. MG-132 (20 μM) treatment for 8 h. Scale bar, 200 μm. **b** Quantification of Cell immunofluorescence in (**a**). **c** Western blot of WCLs and Co-IP samples harvested from HEK-293T cells co-transfected with Myc-BCLAF1 and Flag-SPOP plasmids for 24 h. All data are shown as mean ± SD (n = 3). ***P <

0.001.

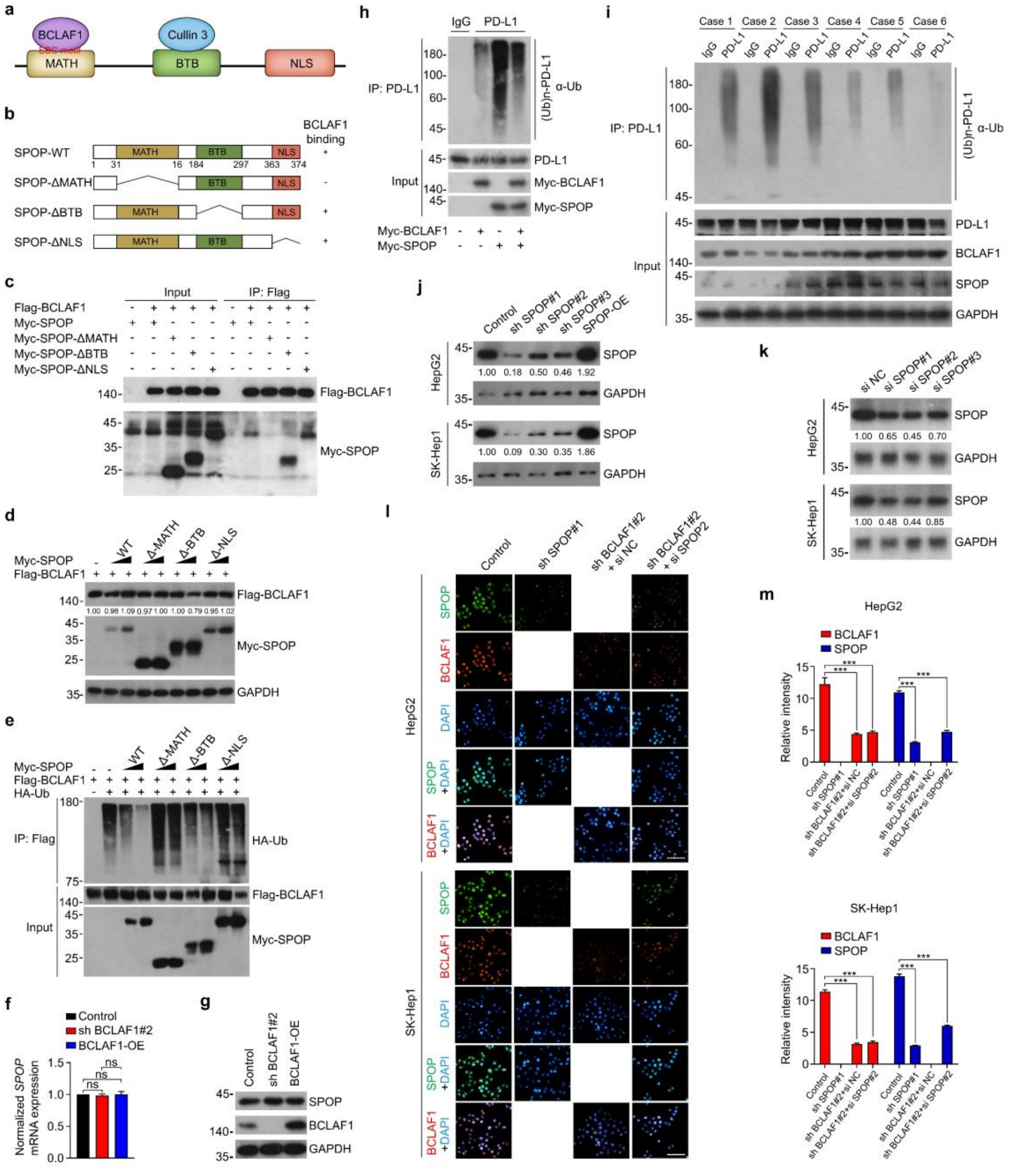

Supplemental Fig. 2 **Identification of the interacting structural domains of BCLAF1 and SPOP, effects of SPOP and BCLAF1 on each other's expression, ubiquitination assay of PD-L1 protein, Western blot of SPOP knockdown, and cellular immunofluorescence to detect BCLAF1 and SPOP expression in HCC cells**

a Diagram of the structure of the SPOP protein. **b** Schematic representation of SPOP deletion mutants. A binding capacity of SPOP to BCLAF1 is indicated with the symbol. **c** Western blot of the indicated proteins in WCLs and Co-IP samples of anti-Flag antibody obtained from HEK-293T cells transfected with indicated plasmids. **d** Western blot of WCLs from HEK-293T cells transfected with indicated plasmids. All quantitation were normalized to the protein levels of GAPDH in Control group. **e** Western blot of the products of *in vivo* ubiquitination assays performed using WCLs from HEK-293T cells transfected with the indicated plasmids. **f** qRT-PCR measurement of *SPOP* mRNA levels in BCLAF1-depleted and BCLAF1-overexpressed SK-Hep1 cells. **g** Western blot of WCLs from BCLAF1-depleted and BCLAF1-overexpressed SK-Hep1 cells. **h** SK-Hep1 cells were treated with MG-132 (20 µM) for 8 h prior to lysis, and *in vivo* ubiquitination assays were performed using the antibodies shown to detect the effect of BCLAF1 on ubiquitination of endogenous PD-L1 mediated by SPOP. **i** Western blot of ubiquitination levels of PD-L1 in HCC tissues. **j** HepG2 and SK-Hep1 cells were infected with lentiviruses expressing SPOP-specific sh RNAs (sh SPOP#1, #2, #3), SPOP-specific overexpression (SPOP-OE), or a control (Control) of the same vector system for 72 h. After infection, WCLs were prepared and SPOP protein levels were determined by Western blot. All quantitation were normalized to the protein levels of GAPDH in Control group. **k** HepG2 and SK-Hep1 cells were transfected with si RNAs specifically targeting SPOP (si SPOP#1, #2, #3) or negative Control (si NC) for 24 h. WCLs were prepared and SPOP protein levels were determined by Western blot. All quantitation were normalized to the protein levels of GAPDH in Control group. **l**

Representative images of Cell immunofluorescence for detection of BCLAF1 and SPOP expression in HepG2 and SK-Hep1 cells. Scale bar, 200 μm. **m** Quantification of Cell immunofluorescence in (**l**). All data are shown as mean ± SD (n = 3). ***$P <$ 0.001, ns $P \geq 0.05$.

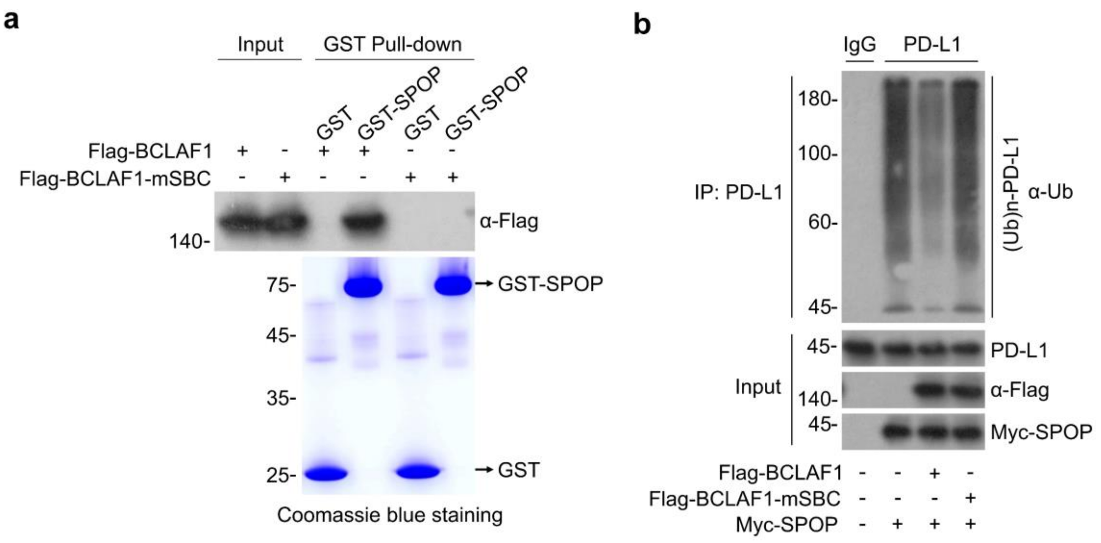

Supplemental Fig. 3 GST pull-down and ubiquitination of endogenous PD-L1

a Bacterially expressed GST-SPOP or GST bound glutathione-Sepharose beads and incubated with bacterially expressed Flag-BCLAF1 or Flag-BCLAF1-mSBC. Bound Flag-BCLAF1 or Flag-BCLAF1-mSBC was detected by Western blot with anti-Flag antibody. GST and GST-SPOP were detected by Western blot and Coomassie blue staining. **b** SK-Hep1 cells were treated with MG-132 (20 μM) for 8 h prior to lysis, and *in vivo* ubiquitination assays were performed using the antibodies shown to detect the effect of BCLAF1-WT and BCLAF1-mSBC on ubiquitination of endogenous PD-L1 mediated by SPOP.

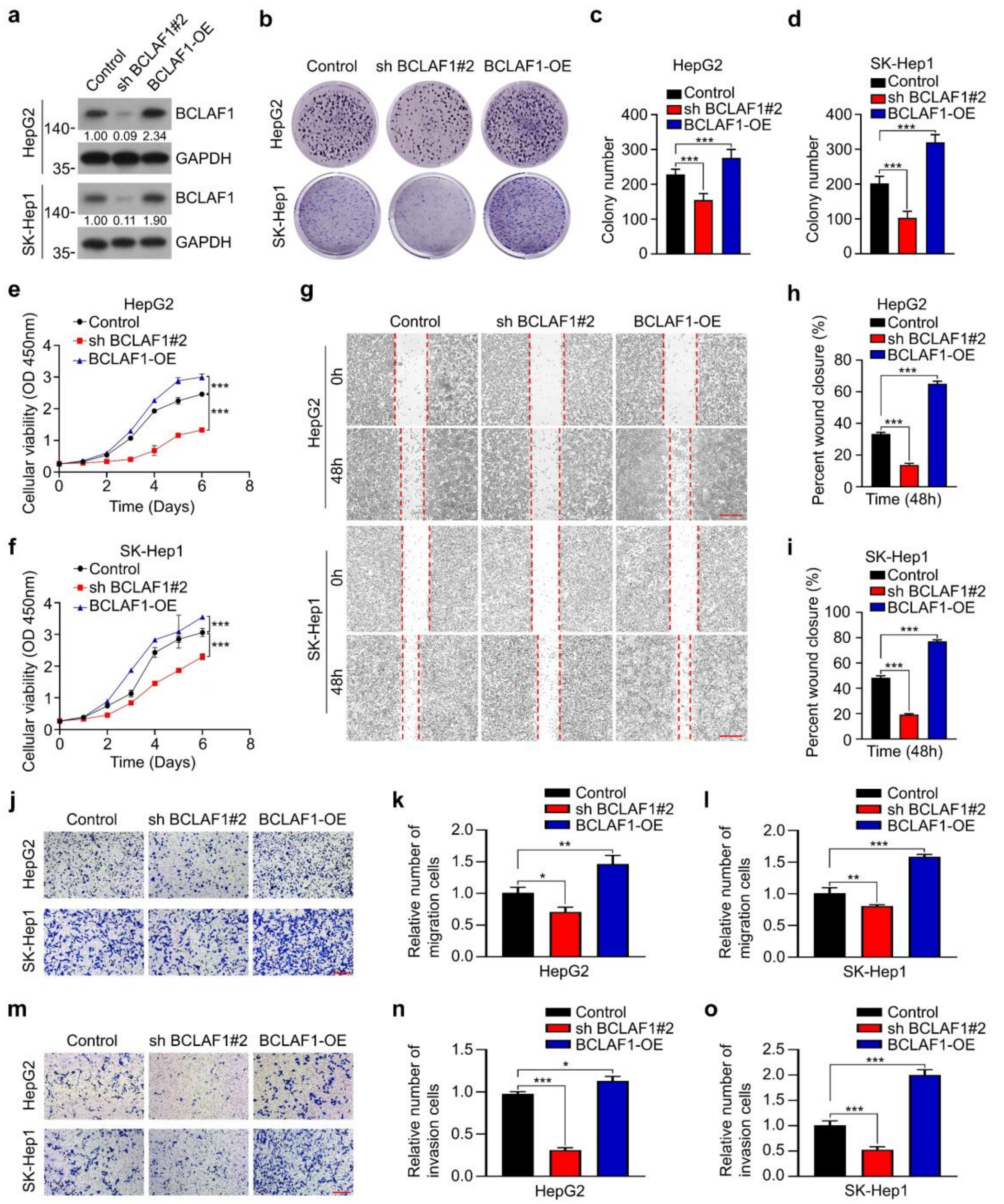

Supplemental Fig. 4 BCLAF1 promotes the proliferation, migration and invasion of HCC cells

a Western blot of WCLs from HepG2 and SK-Hep1 cells infected with lentivirus expressing the indicated sh RNAs and BCLAF1-overexpression. All quantitation were normalized to the protein levels of GAPDH in Control group. **b** Cell colony formation assay of HepG2 and SK-Hep1 cells infected with lentivirus expressing the indicated sh

RNA and BCLAF1-overexpression. **c, d** Statistics of colony number in (**b**). **e, f** Cell proliferation assay of HepG2 cells (**e**) and SK-Hep1 cells (**f**) infected with lentivirus expressing the indicated sh RNA and BCLAF1-overexpression. **g** Wound healing assay of HepG2 and SK-Hep1 cells infected with lentivirus expressing the indicated sh RNA and BCLAF1-overexpression. Scale bar, 800 μm. **h, i** Statistics of the rate of wound healing in (**g**). **j** Cell migration assay of HepG2 and SK-Hep1 cells infected with lentivirus expressing the indicated sh RNA and BCLAF1-overexpression. Scale bar, 200 μm. **k, l** Statistics of the number of cells migrated in (**j**). **m** Cell invasion assay of HepG2 and SK-Hep1 cells infected with lentivirus expressing the indicated sh RNA and BCLAF1-overexpression. Scale bar, 200 μm. **n, o** Statistics of the number of cells invaded in (**m**). All data are shown as mean ± SD (n = 3). $^*P < 0.05$, $^{**}P < 0.01$, $^{***}P < 0.001$.

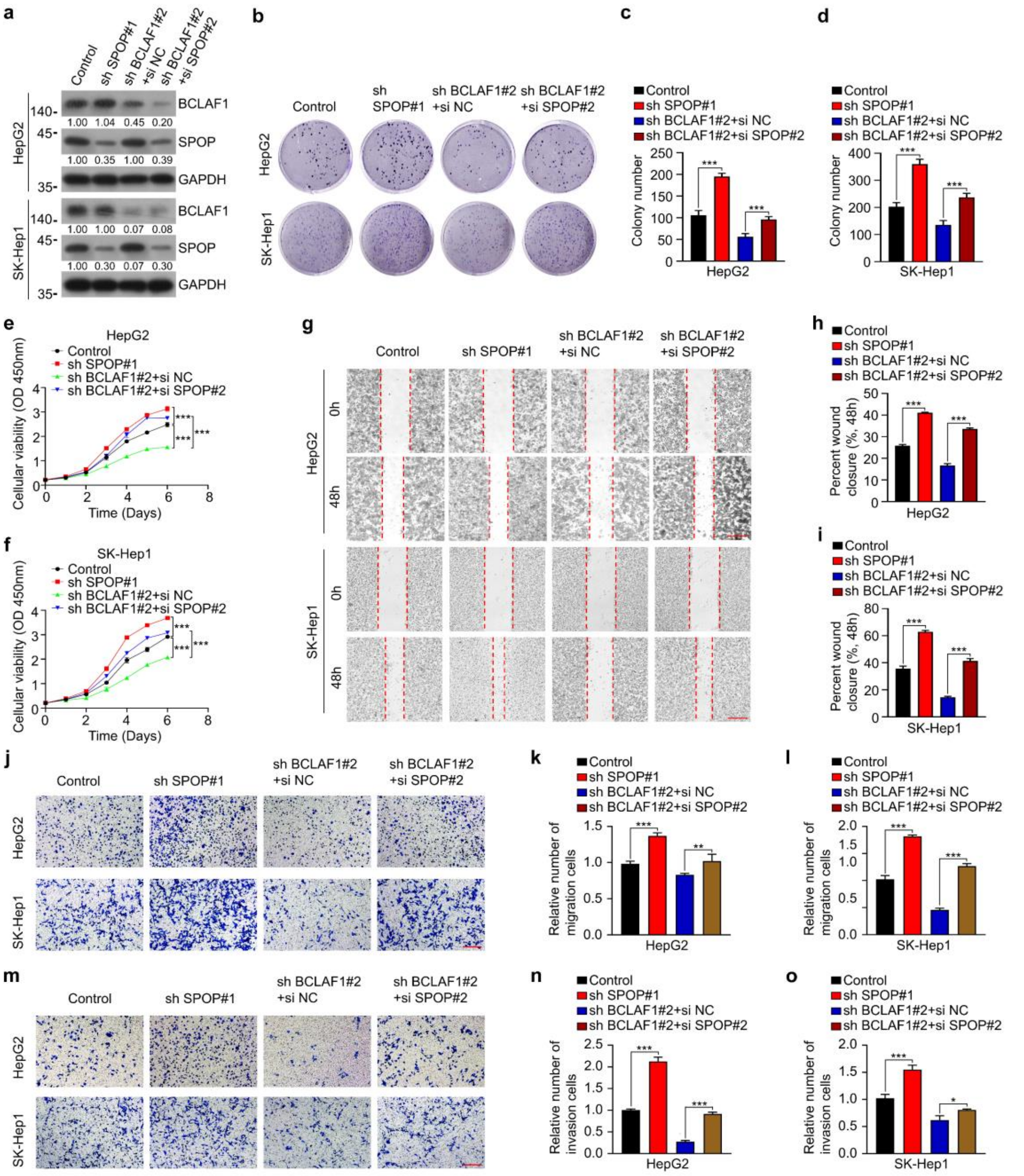

Supplemental Fig. 5 BCLAF1 partly depends on SPOP to participate in the proliferation, migration and invasion of HCC cells

a Western blot of WCLs from HepG2 and SK-Hep1 cells infected with lentivirus expressing the indicated sh RNAs and transfected with the indicated si RNAs. All quantitation were normalized to the protein levels of GAPDH in Control group. **b** Cell colony formation assay of HepG2 and SK-Hep1 cells infected with lentivirus

expressing the indicated sh RNAs and transfected with the indicated si RNAs. **c, d** Statistics of colony number in (**b**). **e, f** Cell proliferation assay of HepG2 cells (**e**) and SK-Hep1 cells (**f**) infected with lentivirus expressing the indicated sh RNAs and transfected with the indicated si RNAs. **g** Wound healing assay of HepG2 and SK-Hep1 cells infected with lentivirus expressing the indicated sh RNAs and transfected with the indicated si RNAs. Scale bar, 800 µm. **h, i** Statistics of the rate of wound healing in (**g**). **j** Cell migration assay of HepG2 and SK-Hep1 cells infected with lentivirus expressing the indicated sh RNAs and transfected with the indicated si RNAs. Scale bar, 200 µm. **k, l** Statistics of the number of cells migrated in (**j**). **m** Cell invasion assay of HepG2 and SK-Hep1 cells infected with lentivirus expressing the indicated sh RNAs and transfected with the indicated si RNAs. Scale bar, 200 µm. **n, o** Statistics of the number of cells invaded in (**m**). All data are shown as mean ± SD (n = 3). **P* < 0.05, ***P* < 0.01, ****P* < 0.001.

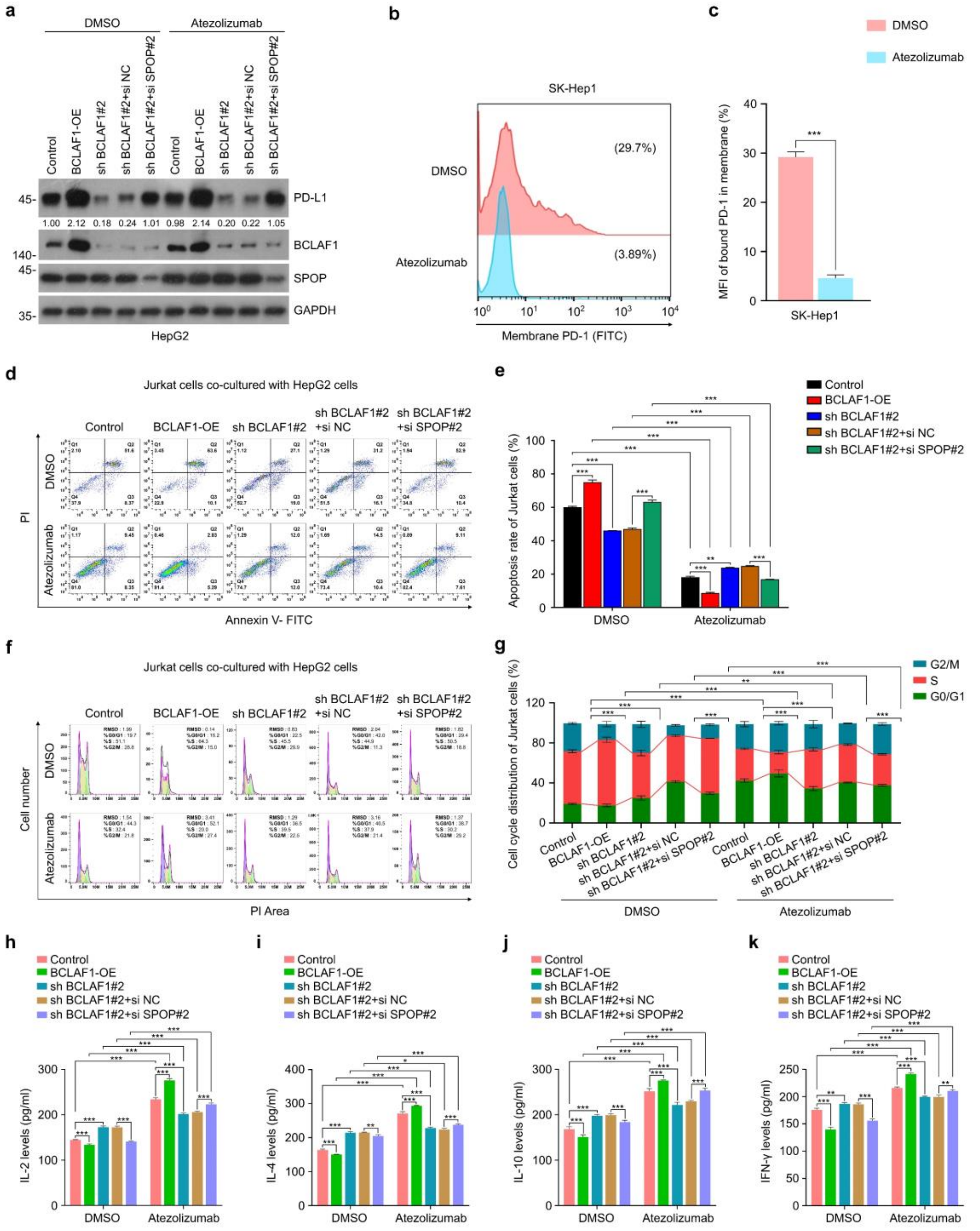

Supplemental Fig. 6 BCLAF1 induces immune escape of HCC cells partly in an SPOP-PD-L1 axis-dependent manner

a HepG2 cells achieving BCLAF1 overexpression, BCLAF1 knockdown, and BCLAF1 knockdown followed by SPOP knockdown were co-cultured with Jurkat cells for 24 h after treatment with DMSO or Atezolizumab (10 ng/mL). Western blot of

HepG2 cells in the HCC cell-Jurkat cells co-culture system for detection of PD-L1 expression levels. All quantitation were normalized to the protein levels of GAPDH in Control group. **b** Flow cytometry analysis of PD-1 binding on the surface of SK-Hep1 cells after treatment of co-culture system with 10 ng/mL Atezolizumab or DMSO for 24 h. **c** Statistics of mean fluorescence intensity (MFI) for PD-1 in (**b**). **d** HepG2 cells achieving BCLAF1 overexpression, BCLAF1 knockdown, and BCLAF1 knockdown followed by SPOP knockdown were co-cultured with Jurkat cells for 24 h after treatment with DMSO or Atezolizumab (10 ng/mL). Apoptosis levels of Jurkat cells were detected by Flow cytometry analysis. **e** Statistics of apoptotic levels of Jurkat cells in (**d**). **f** HepG2 cells achieving BCLAF1 overexpression, BCLAF1 knockdown, and BCLAF1 knockdown followed by SPOP knockdown were co-cultured with Jurkat cells for 24 h after treatment with DMSO or Atezolizumab (10 ng/mL). Cell cycle of Jurkat cells were detected by Flow cytometry analysis. **g** Statistics of cell cycle of Jurkat cells in (**f**). **h-k** HepG2 cells achieving BCLAF1 overexpression, BCLAF1 knockdown, and BCLAF1 knockdown followed by SPOP knockdown were co-cultured with Jurkat cells for 24 h after treatment with DMSO or Atezolizumab (10 ng/mL). The levels of IL-2 (**h**), IL-4 (**i**), IL-10 (**j**), and IFN-γ (**k**) produced by Jurkat cells were detected by ELISA. All data are shown as mean ± SD (n = 3). *$P < 0.05$, **$P < 0.01$, ***$P < 0.001$.

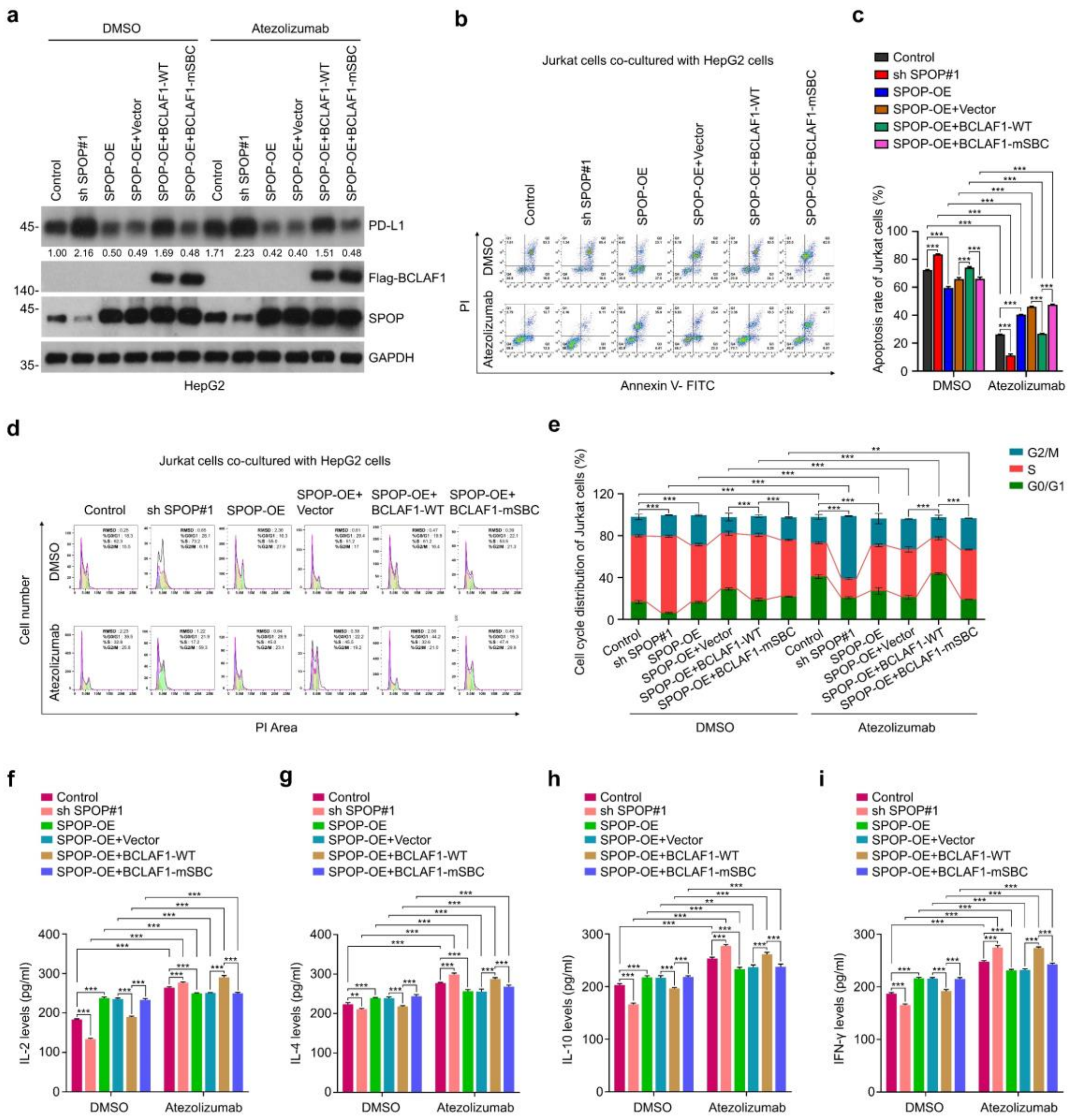

Supplemental Fig. 7 SPOP-binding consensus (SBC) motif-associated BCLAF1 mutant is defective in inducing immune escape of HCC cells

a HepG2 cells achieving SPOP knockdown, SPOP overexpression, and exogenous overexpression of BCLAF1-WT and BCLAF1-mSBC after SPOP overexpression were co-cultured with Jurkat cells for 24 h after treatment with DMSO or Atezolizumab (10 ng/mL). Western blot of HepG2 cells in the HCC cell-Jurkat cell co-culture system for detection of PD-L1 expression levels. All quantitation were normalized to the protein levels of GAPDH in Control group. **b** HepG2 cells achieving SPOP knockdown, SPOP

overexpression, and exogenous overexpression of BCLAF1-WT and BCLAF1-mSBC after SPOP overexpression were co-cultured with Jurkat cells for 24 h after treatment with DMSO or Atezolizumab (10 ng/mL). Apoptosis levels of Jurkat cells were detected by Flow cytometry analysis. **c** Statistics of apoptotic levels of Jurkat cells in (**b**). **d** HepG2 cells achieving SPOP knockdown, SPOP overexpression, and exogenous overexpression of BCLAF1-WT and BCLAF1-mSBC after SPOP overexpression were co-cultured with Jurkat cells for 24 h after treatment with DMSO or Atezolizumab (10 ng/mL). Cell cycle of Jurkat cells were detected by Flow cytometry analysis. **e** Statistics of cell cycle of Jurkat cells in (**d**). **f-i** HepG2 cells achieving SPOP knockdown, SPOP overexpression, and exogenous overexpression of BCLAF1-WT and BCLAF1-mSBC after SPOP overexpression were co-cultured with Jurkat cells for 24 h after treatment with DMSO or Atezolizumab (10 ng/mL). The levels of IL-2 (**f**), IL-4 (**g**), IL-10 (**h**), and IFN-γ (**i**) produced by Jurkat cells were detected by ELISA. All data are shown as mean ± SD (n = 3). $^{**}P < 0.01$, $^{***}P < 0.001$.

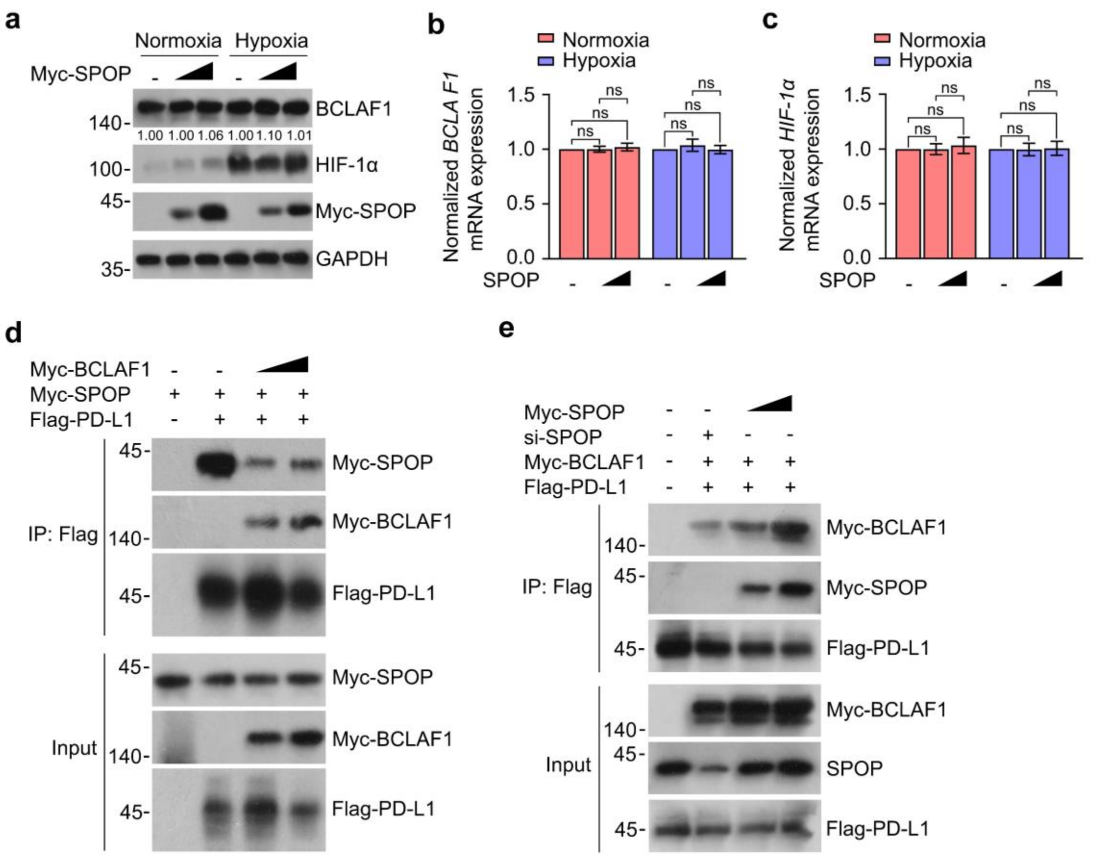

Supplemental Fig. 8 Effect of SPOP on the expression levels of HIF-1α and BCLAF1, the effect of BCLAF1 on SPOP-PD-L1 interaction, and SPOP mediates the indirect interaction between BCLAF1 and PD-L1

a Western blot of WCLs from SK-Hep1 cells treated with 200 μM $Cocl_2$ 24 h. All quantitation were normalized to the protein levels of GAPDH in the untransfected Myc-SPOP plasmid group. **b, c** qRT-PCR measurement of *BCLAF1* (**b**) *and HIF-1α* (**c**) mRNA levels in SK-Hep1 cells treated with 200 μM $Cocl_2$ 24 h. **d, e** Western blot of the indicated proteins in WCLs and Co-IP samples of anti-Flag antibody obtained from HEK-293T cells transfected with indicated plasmids. All data are shown as mean ± SD (n = 3). ns $P \geq 0.05$.

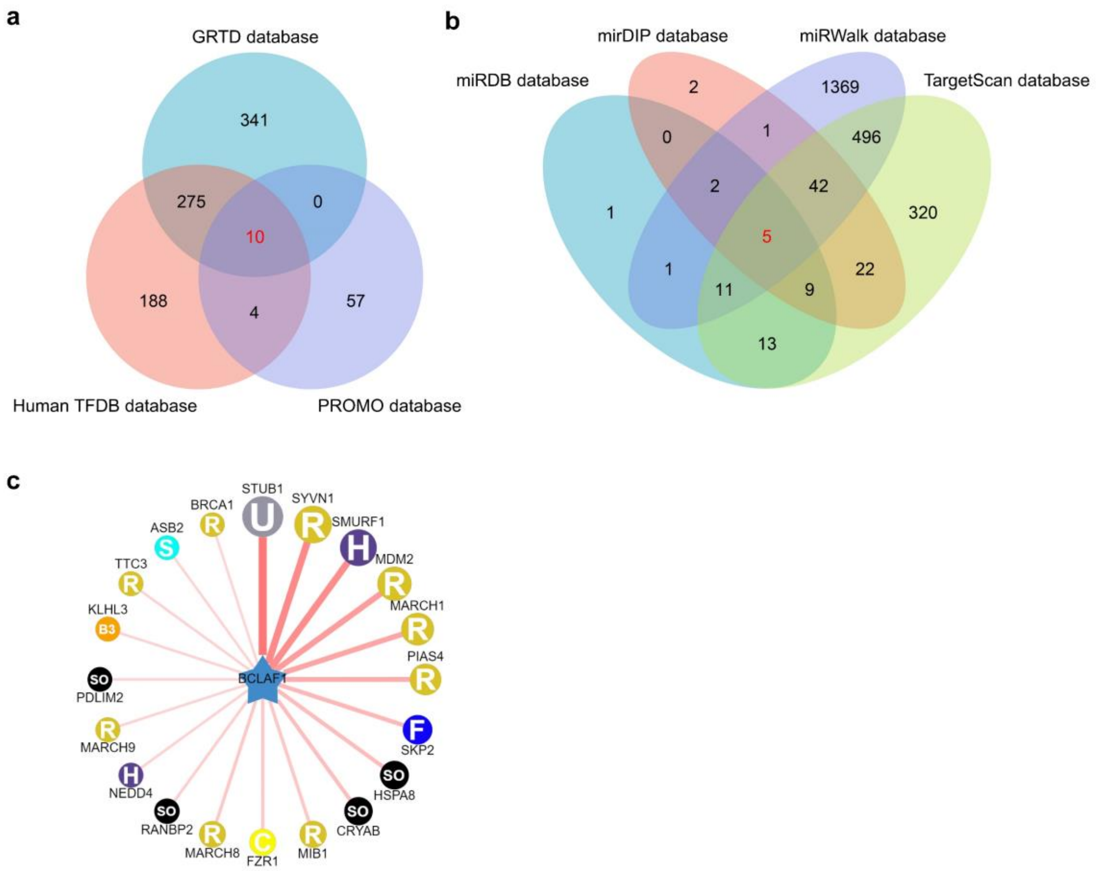

Supplemental Fig. 9 Predicted upstream regulators of BCLAF1 at transcriptional, post-transcriptional, and post-translational levels

a Ten potential transcription factors targeting BCLAF1 predicted by GRTD, HumanTFDB, and PROMO databases. **b** Five potential microRNAs (miRNAs) targeting BCLAF1 predicted by miRDB, mirDIP, miRWalk, and TargetScan databases. **c** Twenty potential E3 ubiquitin ligases targeting BCLAF1 were predicted by the ubibrowser 1.0 database.

Supplemental Table 1. 424 clinical characteristics of HCC patients from TCGA.

Characteristic	Low expression of BCLAF1	High expression of BCLAF1	*P* value
n	187	187	
T stage			0.122
T1	101	82	
T2	46	49	
T3	34	46	
T4	4	9	
N stag			0.622
N0	127	127	
N1	1	3	
M stag			1
M0	138	130	
M1	2	2	
Pathologic stage			0.181
Stage I	96	77	
Stage II	45	42	
Stage III	35	50	
Stage IV	3	2	
Gender			**0.002**
Female	46	75	
Male	141	112	
Age			**0.02**
≤60	77	100	
>60	110	86	
AFP(ng/ml)			**0.011**
≤400	120	95	
>400	24	41	
Vascular invasion			1
No	105	103	
Yes	56	54	

Abbreviations: T, tumor; N, node; M, metastasis; AFP, alpha-fetoprotein.

Supplemental Table 2. Predicted transcription factors targeting *BCLAF1* gene.

Gene symbol	Transcription factor	Position	Binding site
BCLAF1	AR	Promoter	252
BCLAF1	YY1	Promoter	80
BCLAF1	TBP	Promoter	33
BCLAF1	VDR	Promoter	16
BCLAF1	POU2F1	Promoter	14
BCLAF1	POU2F2	Promoter	14
BCLAF1	ATF3	Promoter	11
BCLAF1	STAT4	Promoter	9
BCLAF1	STAT5A	Promoter	7
BCLAF1	WT1	Promoter	5

Supplemental Table 3. Predicted microRNA (miRNA) s targeting *BCLAF1* gene.

Gene symbol	MiRNA	Position	Binding site
BCLAF1	hsa-miR-514b-5p	3' UTR	4
BCLAF1	hsa-miR-513c-5p	3' UTR	4
BCLAF1	hsa-miR-513a-3p	3' UTR	3
BCLAF1	hsa-miR-625-5p	3' UTR	3
BCLAF1	hsa-miR-181a-5p	3' UTR	2

Supplemental Table 4. Predicted E3 ligases targeting BCLAF1 protein.

E3 ligase	Substrate	Confidence score
STUB1	BCLAF1	0.781
SYVN1	BCLAF1	0.746
SMURF1	BCLAF1	0.738
MDM2	BCLAF1	0.71
MARCH1	BCLAF1	0.694
PIAS4	BCLAF1	0.682
SKP2	BCLAF1	0.661
HSPA8	BCLAF1	0.653
CRYAB	BCLAF1	0.649
MIB1	BCLAF1	0.633
FZR1	BCLAF1	0.63
MARCH8	BCLAF1	0.63
RANBP2	BCLAF1	0.617
NEDD4	BCLAF1	0.611
MARCH9	BCLAF1	0.61
PDLIM2	BCLAF1	0.61
KLHL3	BCLAF1	0.61
TTC3	BCLAF1	0.61
ASB2	BCLAF1	0.608
BRCA1	BCLAF1	0.606

Supplemental Table 5. Antibody information.

No	Antibody	Cytokine Species	Cat. No	Source	Application/ Dilutions
1	Anti-PD-L1	Mouse	66248-1-Ig	Proteintech	IB: 1:1000<br>IF:1:50<br>IHC:1:250
2	Anti-BCLAF1	Mouse	67860-1-Ig	Proteintech	IB: 1:8000<br>IF:1:100<br>IHC:1:250
3	Anti-SPOP	Rabbit	16750-1AP	Proteintech	IB: 1:5000<br>IF:1:100<br>IHC:1:250
4	Anti-Flag	Mouse	M185-7	MBL	IB: 1:4000
5	Anti-Myc	Mouse	M192-7	MBL	IB: 1:5000
6	Anti-HA	Mouse	M180-7	MBL	IB: 1:5000
7	Anti-Flag	Mouse	66008-4-Ig	Proteintech	IF: 1:500
8	Anti-HA	Rabbit	81290-1-	Proteintech	IF: 1:250

			RR		
9	Anti-HIF-1α	Rabbit	20960-1-AP	Proteintech	IB: 1:2500
10	Anti-GAPDH	Rabbit	AC001	Abclonal	IB: 1:8000
11	Anti-Mouse	Donkey	AS033	Abclonal	IB: 1:8000<br>IHC:1:200
12	Anti-Rabbit	Donkey	AS038	Abclonal	IB: 1:8000<br>IHC:1:200
13	Anti-Rabbit-488	Donkey	AS035	Abclonal	IF: 1:200
14	Anti-Mouse-Cy3	Goat	AS008	Abclonal	IF: 1:250
15	Anti-Flag beads	Mouse	M2	Sigma	IP
16	Anti-Alexa Fluor 488 dye	Goat	A-11013	Thermo Fisher Scientific	FC
IB: Immunoblot; IF: Immunofluorescence; IHC: immunohistochemistry; IP: Immunoprecipitation; FC, Flow Cytometry					

Supplemental Table 6. Chemicals.

No	Name	Cat. No	Source
1	Atezolizumab	HY-P9904	MedChemExpress
2	MG-132g	S2619	Selleckchem
3	CHX	S7418	Selleckchem
4	Puromycin	S7417	Selleckchem
5	Mitomycin C	GC12353	GLPBIO
6	$Cocl_2$	C804815	Macklin
7	CD3/CD28 T-cell activator	KMS310	Proteintech

Supplemental Table 7. Primers and si RNA/sh RNA sequence information.

**Quantitative RT-PCR primers**		
**Gene**	**F:5'-3'**	**R:5'-3'**
BCLAF1	CGCGTCGAAGGTAG CTCTAT	TTGGAGCGACCCATT TCTTTT
PD-L1	CCTACTGGCATTTGC TGAACGCAT	TTTGCTGAACGCCCC ATACA
HIF-1α	GAAAGCGCAAGTC CTCAAAG	TGGGTAGGAGATGG AGATGC
SPOP	TGACCACCAGGTA GACAGCG	CCCGTTTCCCCCAA GTTA
GAPDH	CATGGCCTTCCGTG TTCCTA	CCCTCAGATGCCTGC TTCA
**Primers for BCLAF1 mutant construction**		
**Gene**	**F:5'-3'**	**R:5'-3'**
BCLAF1-mSBC	TCTGCAGCCGCAGC CGCTTCTCGTTCT	CCTAGAAGATCTATA TGACCGGCGA
**Sequences of sh RNA oligonucleotide**		
**Gene**	**F:5'-3'**	**R:5'-3'**
BCLAF1#1	CACCGCCACTGAAGA	CGGTGACTTCTTGTAT

	ACATAGTACTCGAAAGTACTATGTTCTTCAGTGGC	CATGAGCTTTCATGATACAAGAAGTCACCGAAAA
BCLAF1#2	CACCGCACTTCAGAGTCATTTATTCCGAAGAATAAATGACTCTGAAGTGC	CGTGAAGTCTCAGTAAATAAGGCTTCTTATTTACTGAGACTTCACGAAAA
BCLAF1#3	CACCGCTAGTACACTTGTCCATTCTCGAAAGAATGGACAAGTGTACTAGC	CGATCATGTGAACAGGTAAGAGCTTTCTTACCTGTTCACATGATCGAAAA
SPOP#1	CACCGGATTTCATCAACTATCATGCCGAAGCATGATAGTTGATGAAATCC	CCTAAAGTAGTTGATAGTACGGCTTCGTACTATCAACTACTTTAGGAAAA
SPOP#2	CACCGCAGTGGATTTCATCAACTATCGAAATAGTTGATGAAATCCACTGC	CGTCACCTAAAGTAGTTGATAGCTTTATCAACTACTTTAGGTGACGAAAA
SPOP#3	CACCGCGCTTAAAGGTCATGTGTGACGAATCACACATGACCTTTAAGCGC	CGCGAATTTCCAGTACACACTGCTTAGTGTGTACTGGAAATTCGCGAAAA
**Sequences of si RNAs**		
**Gene**	**F:5'-3'**	**R:5'-3'**
SPOP#1	ACUCAGGCAGUGGAUUUCAUCAACU	AGUUGAUGAAAUCCACUGCCUGAGU
SPOP#2	GGGCUUCUCCCUGAUGACAAGCUUA	UAAGCUUGUCAUCAGGGAGAAGCCC
SPOP#3	GGGCAUAUAGGUUUGUGCAAGGCAA	UUGCCUUGCACAAACCUAUAUGCCC
